# Cognitive Improvement during Treatment for Mild Alzheimer’s Disease with a Chinese Herbal Formula: A Randomized Controlled Trial

**DOI:** 10.1371/journal.pone.0130353

**Published:** 2015-06-15

**Authors:** Yulian Zhang, Cuiru Lin, Linlin Zhang, Yuanwu Cui, Yun Gu, Jiakui Guo, Di Wu, Qiang Li, Wanshan Song

**Affiliations:** 1 Department of Acupuncture and Cerebropathy, Second Affiliated Hospital of Tianjin University of Traditional Chinese Medicine, Tianjin, China; 2 Department of Geriatric, Longhua Hospital of Shanghai University of Traditional Chinese Medicine, Tianjin, China; 3 Department of Traditional Chinese Medicine, Tianjin Huanhu Hospital, Tianjin, China; 4 Graduate Institutes, Tianjin University of Traditional Chinese Medicine, Tianjin, China; National Health Research Institutes, TAIWAN

## Abstract

**Objectives:**

To explore the efficacy of Chinese herbal formula compared with donepezil 5mg/day in patients with mild Alzheimer’s disease (AD).

**Methods:**

Patients with mild AD meeting the criteria were randomized into Chinese herbal formula Yishen Huazhuo decoction (YHD) group and donepezil hydrochloride (DH) group during the 24-week trial. The outcomes were measured by ADAS-cog, MMSE, ADL, and NPI with linear mixed-effect models.

**Results:**

144 patients were randomized. The mean scores of ADAS-cog and MMSE in both YHD group and DH group both improved at the end of the 24-week treatment period. The results also revealed that YHD was better at improving the mean scores of ADAS-cog and MMSE than DH. Linear mixed-effect models with repeated measures showed statistical significance in time × group interaction effect of ADAS-cog and also in time × group interaction effect of MMSE. The data showed YHD was superior to DH in improving the scores and long term efficacy.

**Conclusions:**

Our study suggests that Chinese herbal formula YHD is beneficial and effective for cognitive improvement in patients with mild AD and the mechanism might be through reducing amyloid-β (Aβ) plaque deposition in the hippocampus.

**Trial Registration:**

Chinese Clinical Trial Registry ChiCTR-TRC-12002846

## Introduction

Alzheimer’s disease (AD), a complex neurodegenerative disease, is the major cause of dementia in the elderly, affecting more than 25 million people worldly [1]. AD is characterized by a progressive and irreversible deterioration of cognitive and functional abilities, leading to complete dependence. The cause for most cases is still unknown, and there exist several hypotheses to explain it, such as genetic heritability [2, 3], cholinergic hypothesis [4], amyloid hypothesis [5], Tau hypothesis [6], and other hypotheses [7, 8].

At present, in Europe, the United States, and Asia, the mainstream medication for AD is typically acetylcholine esterase inhibitor (AchEI), such as donepezil hydrochloride (DH) [9]. Though it has shown significantly clinical efficacy and safety in the treatment of AD, symptomatic improvement is limited in magnitude and duration, after which patients continue to decline [10]. Therefore, new therapeutic agents are essential.

Natural products have been used for medicine for a long time, and much research effort has been made to develop the anti-AD agents from natural sources [11]. Traditional Chinese Medicine (TCM) has a long history of preventing and treating cognitive decline [12] and the efficacy of Chinese herbs to improve cognitive function has been studied in many trials [13–16]. Icariin(C33H40O15; molecular weight 676.65) is a flavonoid isolated from Epimedii herba (Yinyanghuo) and it is also the major pharmacological active component of Epimedii herba, a traditional Chinese herb widely used as a tonic and antirheumatic remedy. It has been reported before that icariin has vasodilatory and cardioprotective effects. Recently it was reported that icariin could improve the spatial learning and memory abilities in aluminum-intoxicated rats and decrease the level of amyloid-β (Aβ) in the hippocampus of aluminum-intoxicated rats [17]. Moreover, icariin could improve the learning and memory abilities in Aβ25–35-induced Alzheimer's disease rats through decreasing the production of insoluble fragments of Aβ [18].

Tetramethylpyrazine (TMP, C8H12N2; molecular weight 136.19), a major component of a Chinese herb Ligusticum wallichi Franchat (chuanxiong), has been widely used in the treatment of cerebrovascular diseases. Also, several studies have demonstrated that TMP could improve learning and cognitive function, through inhibiting calcium overload, anti-apoptotic activity, and anti-inflammatory potential [19, 20]. A few reports showed that Astragali Radix(Huangqi) extract could affect brain function. For example, astragalosides, the major components of Astragali Radix, could improve memory in aged mice [21]. Also, extracts of Astragali Radix could ameliorate the memory deficit in mice caused by Ab25–35 [22].

Therefore, our group developed a Chinese herbal formula Yishen Huazhuo decoction(YHD) to treat mild AD, which is composed of Yinyanghuo (Epimedium), Nvzhenzi(Fructus Ligustri Lucidi),Buguzhi (Psoralea fruit), Heshouwu (Radix Polygoni Multiflori), Huangqi (Radix Astragali), Chuanxiong(Ligusticum wallichi Franchat), and Shichangpu(Acorus gramineus). Our previous pharmacological studies have shown that the formula can improve the cognitive function of mice with AD.

Here, we conducted a 24-week, randomized, double-blind, double-dummy, and multicenter trial to explore the efficacy and safety of the Chinese herbal formula in improving cognitive function in patients with mild AD.

## Methods and Materials

We did the 24 weeks, randomized, double-blind, double-dummy, DH-controlled and multicenter trial (ChiCTR-TRC-12002846), which was conducted between May 1st, 2011 and May 30th, 2014 in three centers, the Second Affiliated Hospital of Tianjin University of TCM, Tianjin Huanhu Hospital, and Longhua Hospital of Shanghai University of TCM, and was approved by the Ethics Committee of the Second Affiliated Hospital of Tianjin University of TCM in July 26th, 2011(S1 Fig). The trial was performed according to the principles of the Declaration of Helsinki.

We confirm that all ongoing and related trials for this drug/intervention are registered. The trial is funded by Ministry of Science and Technology of China and was one of the 973 Program (2010CB530405). Due to time limitations, we started the recruitment and the registration simultaneously in May, 2011. We tried to register the study on the U.S. Food and Drug Administration (FDA), but failed to get access to it after months of effort. We then decided to register on the Chinese Clinical Trial Registry in March 2012 and after months of complex procedures, we finally succeeded.

### Patients

Patients were enrolled from outpatient clinics. Inclusion criteria for this study were: 1) a diagnosis of dementia of the Alzheimer’s type according to the Diagnostic and Statistical Manual of Mental Disorders, 4th edition (DSM-IV; APA, 1994; provided in S2 Trial Protocol (English)). The diagnosis was confirmed by image test (CT/MRI) and diagnosis procedures among three hospitals were standardized; 2) women or men aged 50–85 years; 3) Hachinski Ischemic Score (HIS) ≤4; 4) Hamilton Depression Rating Scale (HAMD) score of ≤7; 5)written informed consent from all patients (or their legal representatives); 6) Clinical Dementia Rating (CDR) score of 1.

Exclusion criteria included: 1) with vascular dementia or any neurological disorder other than AD that contributed substantially to dementia; 2) with severe heart, liver, kidney and blood system diseases (sinus bradycardia and atrioventricular block, AST and ALT 2 times more than the upper limit of normal values; kidney function tests showing BUN 1.5 times higher than the upper limit of normal values, Cr more than the normal values); 3)allergies or allergic to donepezil hydrochloride and Piperidine Derivatives; 4) the use of any drugs that may affect cognitive function 4 weeks prior to randomization; 5) uncontrolled hypertension; 6) with diseases that may interfere with cognitive tests such as aphasia, hemiplegia, and others(severe visual or hearing loss); 7) with serious complications (asthma and chronic obstructive pulmonary disease(COPD)); 8) participation in another investigational new drug trial; 9) with advanced, severe disease that could interfere with study assessments.

### Study design

Due to the differences in appearance of the drugs, dosage, and administration methods between YHD and DH, we used a double-blind and double-dummy design. The simulations were composed of amylum. YHD-simulation, produced and quality controlled by Shenzhen Sanjiu Modern Chinese Medicine Limited Company (Tianjin, China), has the same package, color, smell with YHD; DH-simulation, produced by Eisai China Inc. (Tianjin, China), has the same package, appearance, color, and taste with DH. Patients in both groups should take drugs and simulations simultaneously.

Selection for a treatment group was determined by a computer-generated randomization list under the help of a professional statistician, in a 1:1 ratio in all three centers, and the random number table was sealed in a special envelope. The researchers, drug administrators, and patients were all unaware of the blind design. The evaluators and the statisticians were not involved in the trial.

### Drug administration

The TCM formula in our study is YHD, including Yinyanghuo (Epimedium) 10g, Nvzhenzi(Fructus ligustri lucidi) 10g, Buguzhi (Psoralea fruit) 10g, Heshouwu (Radix polygoni multiflori)10g, Huangqi (Radix astragali) 10g, Chuanxiong(Ligusticum wallichi franchat) 6g, and Shichangpu(Acorus gramineus) 6g in one unit(S1 Table). Each herb was provided as herbal concentrate-granules in one bag (the procedure is in S1 Methods and Materials) and quality controlled by Shenzhen Sanjiu Modern Chinese Medicine Limited Company (Tianjin, China). Aristinic acid is not included in YHD decoction. Every patient in YHD group orally took 100 ml of the decoction once a day half an hour after breakfast and DH-simulation 5 mg before sleep for 24 weeks. Patients in DH group orally took DH, provided by Eisai China Inc. (Tianjin, China) 5 mg each day before sleep, and YHD-simulation 100 ml of the decoction half an hour after breakfast for 24 weeks. The lot number of Chinese herbs, DH, and the simulations are provided in S2 Table.

### Assessment

The primary outcomes were the Alzheimer’s Disease Assessment Scale-cognitive subscale (Chinese version) (ADAS-cog) and Mini-Mental State Examination (Chinese version) (MMSE) measured at week 0, 12, 24, and 48. The ADAS-cog, a widely used cognitive assessment instrument in AD clinical trials, consists of memory, language, orientation, and praxis assessments. Its scores range from 0 to 70, with higher values indicating higher degree of deficit. MMSE, commonly used in medicine to screen for dementia and to estimate the severity of cognitive impairment, includes simple questions in a number of areas: the time and place of the test, repeating lists of words, language use and comprehension, and basic motor skills. Any score greater than or equal to 27 points (out of 30) indicates a normal cognition. Below this, scores can indicate severe (≤9 points), moderate (10–18 points) or mild (19–24 points) cognitive impairment [23].

The secondary outcome measurements included the Activity of Daily Living Scale (Chinese version) (ADL), a 20-item questionnaire designed to measure the patient’s ability to carry out daily activities such as medication management, food preparation, personal hygiene, and transportation utilization, with each item rating between 0 and 10; and the Neuropsychiatric Inventory (Chinese version) (NPI) to assess the severity of symptoms in 10 behavioral domains which includes delusions, hallucinations, agitation/aggression, dysphoria, anxiety, euphoria, apathy, disinhibition, irritability/lability, and aberrant motor behavior. The total score ranges from 0 to 120, where higher values denote higher severity of symptoms [24, 25].

The safety parameters including spontaneously reported adverse events (AEs) or serious AEs (SAEs), vital signs (temperature, heart rate and blood pressure), physical examination, and laboratory tests were assessed during each visit.

### Sample size

According to the reported trial [26], a decrease of 0.67±6.29 (Mean±SD) in ADAS-cog score could be measured after the 24-week treatment by DH 5mg/d. Normally, non-inferiority margin should be less than 1/2 (50%) of the standard deviation [27], thus we set δ as 1/4 (25%) of the standard deviation. With α at 0.05, β = 0.2 (80% power), δ = 1.5, and s = 4, we need 89 patients in each group. Assuming 15% attrition, this inflated the sample size to 103 per group. However, considering the numbers of outpatients in three centers, long term trial, and expenditures, after consulting clinical doctors and professional statistician, we decided to enroll 150 patients, 50 from the Second Affiliated Hospital of Tianjin University of TCM, 30 from the Tianjin Huanhu Hospital, and 70 from the Longhua Hospital of Shanghai University of TCM.

### Statistical analysis

Statistical analyses for our trial were performed using the Statistical Package for the Social Sciences (SPSS) v19.0 software. Statistical testing was two-sided and p < 0.05 was considered statistically significant. Descriptive statistics were presented for the baseline characteristics. Chi square test was applied to comparable count data, and independent samples t test to measurement data. Efficacy outcome parameters were analyzed on an intent-to-treat (ITT) basis. The missing data were not disposed. Linear mixed-effect models were used to analyze repeated measures; group and time were entered as fixed effects while group ×time as interaction effect; the measurement indexes were dependent variables and the patients were random variables. The underlying covariance structure was **UN** covariance structure. Two-independent-samples t test was referred to when the difference met normal distribution.

All AEs were coded using a World Health Organization (WHO)-based dictionary of preferred terms. The numbers (percentages) of patients with at least one AE, at least one serious AE (SAE), and at least one AE leading to discontinuation were summarized irrespective of relationship to treatment. All safety-related observations were summarized using descriptive statistics.

## Results

**Fig 1:**
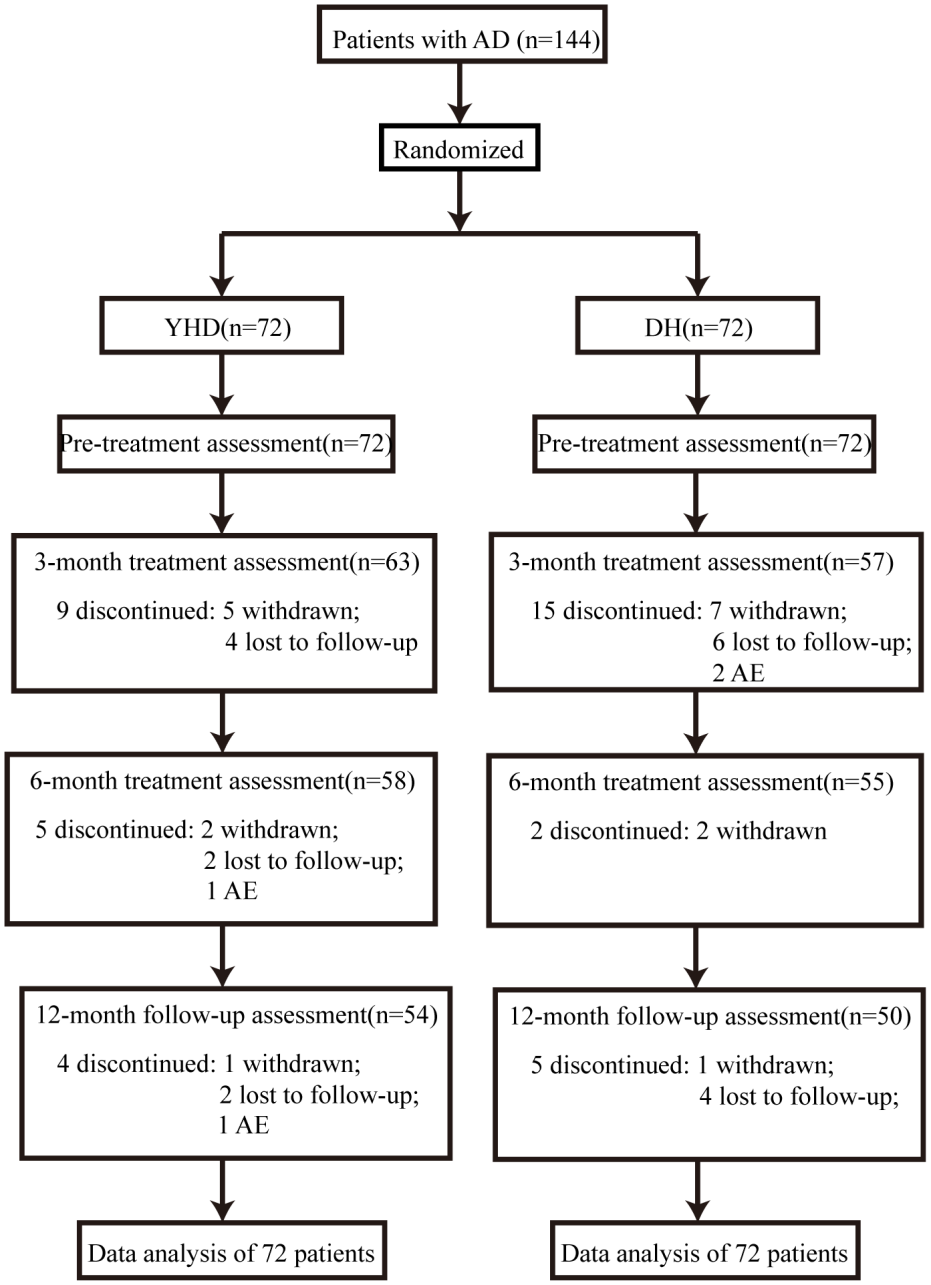
Flow diagram and disposition of the two groups.

The first patient was screened in May 17th, 2011 and the last patient completed the study in May 30th, 2014. A total of 144 patients were enrolled into the trial (Fig 1), 48 from the Second Affiliated Hospital of Tianjin University of TCM, 24 from the Tianjin Huanhu Hospital, and 72 from the Longhua Hospital of Shanghai University of TCM. There was no statistically significant difference at baseline between the patients in the two groups (Table 1). A total of 104 patients (72.2%) completed the study, 54 (75%) in the YHD group and 50(69.4%) in the DH group.

**Table 1:** Baseline demographics and disease characteristics.

	**YHD** (n = 72)	**DH** (n = 72)	*P*
**Gender**			
Male n(%)	26(36.1)	29(40.3)	0.607
Female n(%)	46(63.9)	43(59.7)	
**Age, years**			
(Mean±SD)	72.79±6.76	72.97±6.59	0.871
Range	61.00~85.00	60.00~84.00	
**Education level**			
Illiteracy n(%)	6(8.3)	10(13.9)	0.686
Primary school n(%)	18(25.0)	20(27.8)	
Middle school n(%)	34(47.2)	28(38.9)	
College n(%)	9(12.5)	7(9.7)	
Academic high school n(%)	5(6.9)	7(9.7)	
**Family status**			
Married n(%)	64(88.9)	61(84.7)	0.527
Widowed n(%)	7(9.7)	10(13.9)	
Divorced n(%)	1(1.4)	0(0)	
Remarried n(%)	0(0)	1(1.4)	
**Occupation**			
Workers n(%)	36(50.0)	37(51.4)	0.146
Farmers n(%)	1(1.4)	2(2.8)	
Officials n(%)	1(1.4)	0(0)	
Staffers n(%)	8(11.1)	3(4.2)	
Cadres n(%)	2(2.8)	9(12.5)	
Teachers n(%)	6(8.3)	2(2.8)	
Health workers n(%)	2(2.8)	2(2.8)	
Freelancers n(%)	1(1.4)	0(0)	
Others n(%)	15(20.8)	17(23.6)	
**Ethnics**			
Han	72	72	
Others	0	0	
**Alzheimer’s disease duration**			
**months**(Mean±SD)^a^	28.75±14.22	26.72±13.23	0.377

Notes:

*a* indicates time since Alzheimer’s disease was first diagnosed by a physician.

### The major components of YHD decoction

There were eight major categories of compounds in the YHD decoction. Flavones (icariin, epimedin C, ligustroside), anthraquinones (emodin, physcion, emodin-8-o-β-D-glucoside, physcion-8-o-β-D-glucoside), polyhydroxy phenols (2,3,5,4’-tetrahydroxy stilbene-2-o-glycoside TSG), triterpenoid sapnins (oleanolic acid, astragaloside I, astragaloside II, astragaloside III, isoastragaloside I, isoastragaloside II), monoterpenes (linalool), phenylpropanoids (α asarone, β asarone), coumarin (psoralen, isopsoralen), and alkaloids (tetramethylpyrazine TMP) were in the decoction, among which icariin, TSG, β asarone, astragalosides, and TMP were the major components (S2 Fig).

### Outcome Measures

The mean±SD scores of both primary and secondary outcomes (including ADAS-cog, MMSE, ADL, and NPI) of the patients in the two groups are shown in Table 2.

**Table 2:** Mean efficacy scores at all time points and change at week 24, and 48 (Mean±SD).

	Group	Baseline	12weeks	24weeks	48weeks	Change 24 weeks	Change 48 weeks
**ADAS-cog**	**YHD**	22.03±9.14	20.63±9.37*	18.18±9.79*	19.65±10.62*	-3.10±4.55*	1.45±3.74*
	**DH**	24.62±8.57	24.01±8.64	22.24±8.89	25.16±11.21	-1.22±4.99	4.13±8.62
**MMSE**	**YHD**	20.49±4.29	21.78±4.88	22.67±5.40	22.37±5.31*	1.72±2.59	-0.40±1.63*
	**DH**	19.82±3.54	21.14±4.13	21.53±3.93	20.60±4.52	1.27±2.23	-1.38±2.68
**ADL**	**YHD**	28.81±7.36	27.68±6.48	27.00±5.68	28.00±5.35	-1.12±3.16	1.36±3.60
	**DH**	29.72±5.56	29.46±5.96	28.82±6.14	30.46±6.75	-1.07±3.86	2.38±5.08
**NPI**	**YHD**	1.50±2.96	-	0.68±1.34	0.63±1.96	-0.71±2.44	-0.11±2.17
	**DH**	1.35±2.04	-	1.22±1.99	1.20±2.07	-0.54±1.65	-0.02±1.55

* indicates significance between the two study groups.

**Fig 2:**
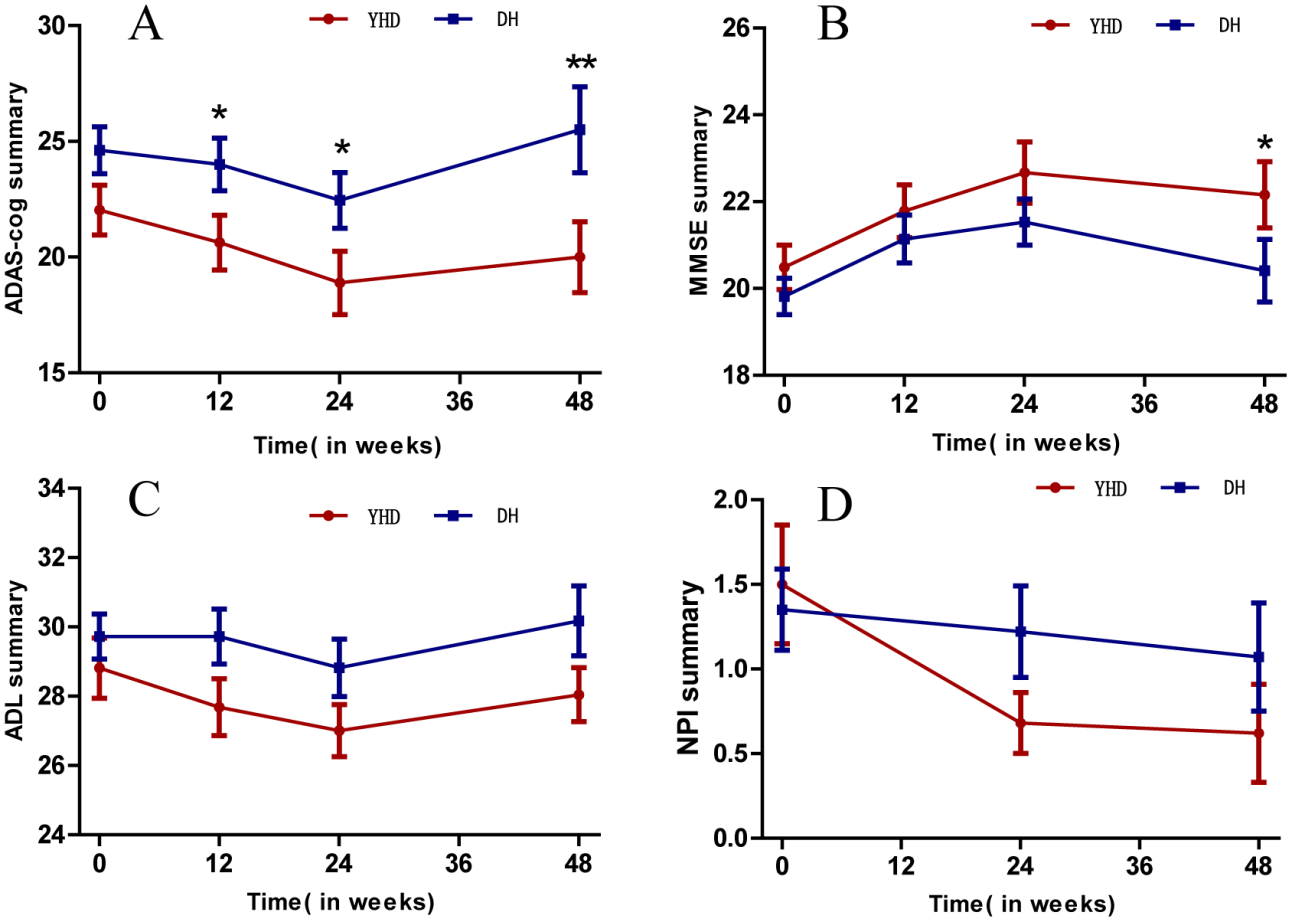
Scores of ADAS-cog, MMSE, ADL, NPI in YHD and DH group. A. ADAS-cog scores in groups of YHD and DH; B. MMSE scores in groups of YHD and DH; C. ADL scores in groups of YHD and DH; D. NPI scores in groups of YHD and DH.

Linear mixed-effect models with repeated measures showed interaction of ADAS-cog and MMSE scores during the 24-week treatment, but no interaction of ADL and NPI. A significant time × group interaction effect was observed in ADAS-cog (F110.186 = 3.164, p = 0.027) (Table 3); the scores in YHD group significantly reduced in week 12 and 24, while the scores of DH group declined in week 24(p = 0.061). The mean scores of ADAS-cog showed a decrease of 3.10±4.55 in YHD group and a decrease of 1.22±4.99 in DH group during the 24 weeks treatment, with statistical significance. The follow-up at week 48 showed that after the treatment, compared with week 24, the scores of ADAS-cog increased 1.45±3.74 in YHD group, but less than the increase of 4.13±8.62 in DH group (p<0.05) (Fig 2).

**Table 3:** Linear mixed effect model estimates of fixed effects (ADAS-cog, MMSE, ADL, NPI).

**Effect**	**F test**	**Df**	***p-value***
**ADAS-cog**			
Group	7.960	133.870	0.006
Time	12.771	110.186	P<0.001
Group×Time	3.164	110.186	0.027
**MMSE**			
Group	2.770	141.120	0.098
Time	18.115	107.776	P<0.001
Group×Time	3.251	107.776	0.025
**ADL**			
Group	1.944	125.621	0.166
Time	6.522	99.309	P<0.001
Group×Time	1.083	99.309	0.360
**ADL** *			
Group	1.943	134.117	0.166
Time	6.612	93.219	P<0.001
**NPI**			
Group	0.609	142.241	0.437
Time	4.425	124.376	0.014
Group×Time	1.077	124.376	0.344
**NPI** *			
Group	1.278	134.535	0.260
Time	4.510	125.039	0.013

* indicates the main effects after dropping the insignificant group x time interaction.

Time × group interaction effect was observed in the scores of MMSE (F107.776 = 3.251, p = 0.025). The scores in both groups increased in week 12 and 24, by 1.72±2.59 in YHD group and 1.27±2.23 in DH group, but there is no significant difference between groups during the 24-week treatment. Compared with week-24, the scores of ADAS-cog in the follow up in week 48 showed a decrease of 0.40±1.63 in YHD group, but significantly less than the decrease of 1.38±2.68 in DH group (p<0.05).

Both YHD and DH could improve the scores of ADL and NPI, but without statistically significant difference between groups. After dropping the insignificant group × time interaction, there was still no statistically significant difference between groups.

### Safety

There were no statistically significant differences between the two groups with respect to the incidence of adverse events. There were 5 REs in YHD group and 6 in the other group (Table 4) and most AEs were mild, most frequent in diarrhea (2) and catching cold (2). Both YHD group and DH group reported a cerebral infarction, but they were not considered to be related to the treatment. Adverse events caused two drop-outs (2.8%) in the DH group and two (2.8%) in the YHD group.

**Table 4:** Reported adverse events.

	**YHD** n(%)	**DH** n (%)
Diarrhea	0 (0%)	2 (33.33%)
Cerebral infarction	1 (20%)	1 (16.67%)
Catching cold	1 (20%)	1 (16.67%)
Abnormal LFTs	1 (20%)	1 (16.67%)
Arthralgia	1 (20%)	0 (0%)
Constipation	1 (20%)	0 (0%)
Insomnia	0 (0%)	1 (16.67%)

Notes: LFTs indicates liver function tests.

There was no clinically significant change in patients of the two groups in physical examination and vital signs during the study.

## Discussion

Donepezil is an established, well-received and effective drug, approved for the treatment of mild to moderate and severe AD [28]. It is available to increase cognitive function as measured by the ADAS-cog [29]. Considering that its subsequent improvement after the treatment has been reported to decrease [30,31], we tried to use Chinese herbal formula to treat mild AD based on the long term use in preventing and treating cognitive decline.

In this study, we assessed the impact of Chinese herbal formula YHD on patients with mild AD compared with DH at 5 mg/day. We speculate that YHD may function in attenuating Aβ neurotoxicity and reducing the Aβ plaque deposition in the hippocampus which is a key pathological event in AD. In this formula, icariin, TSG, β asarone, astragalosides, and TMP are the major components. Icariin, the major component extracted from Epimedium, has been reported to have cardiac protective effects [32], anti-inflammatory effects [33], and even anti-tumor properties [34]. Also, it has been repeatedly shown that icariin has effects on transgenic models of AD. It can attenuate Aβ neurotoxicity [35], reduce the Aβ burden and amyloid plaque deposition in the hippocampus [36], and also has positive effects on cognition [37], learning, and memory [38, 39]. Experimental evidence indicates that β-asarone, the major ingredient of Acorus gramineus, has neuroprotective effects. It can also ameliorate impairment of learning and memory in rats by antagonizing neurotoxicity of Aβ [40, 41]. TMP may be useful in ameliorating microglia-mediated inflammatory by Aβ [42], and studies also suggest it could remarkably enhance learning and memory in AD mice model [43]. TSG has exhibited neuroprotection against brain ischemic injury [44] and can improve cognitive deficits in aged rats [45] according to several studies. Furthermore, the protective effects of TSG on learning and memory deficits in transgenic mice [46], and in Aβ-induced AD mice [47] have been reported.

The results show that YHD can improve cognitive function according to the scores of ADAS-cog, MMSE, ADL, and NPI. Moreover, Chinese herbal formula YHD shows statistically significant superiority over DH 5mg/day in improving the scores of ADAS-cog at all time points during the 24-week treatment, demonstrating evidence of better efficacy. Also, at the follow up in week 48, the scores of YHD measured by ADAS-cog and MMSE show less deterioration than DH, which suggests a possible longer-term symptomatic benefit.

The ADAS-cog is the best suitable for assessing mild-to-moderate stages of dementia [48, 49]. In this study, the ADAS-cog score shows a better improvement in the YHD group than that in the DH group. However, we cannot say that YHD is better than DH in changing cognitive function. Donepezil hydrochloride administrated in our study was at 5 mg/day, an approved dosage for the treatment of mild to moderate AD. It has already been confirmed that the efficacy of donepezil is dose-dependent [50–52]. In earlier studies, the ADAS-cog score showed significant improvement at donepezil 23 mg/d or 10 mg/d compared with 5mg/d [53–56].

Both YHD and DH are well-tolerated. Cases of cerebral infarction in the YHD group, catching cold in both groups and constipation in DH group are not considered to be related to the drugs. Due to the small sample, our results could not provide significant evidence to prove which drug is better in safety.

It should be noted that our study have some limitations. First, the small sample size leads to underpowered results. Second, our study lack control group with higher dosage of donepezil hydrochloride such as 10mg/day and a placebo control group to exclude the influence from a possible large placebo effect to get further comparison. Third, it is uncertain whether the findings in Han Chinese can be extrapolated to other populations.

## Conclusions

Our study suggests that Chinese herbal formula YHD is beneficial and effective for improvement in cognitive deficits in patients with mild AD, and the mechanism might be by reducing the Aβ plaque deposition in the hippocampus. If larger sample size studies could confirm the findings, we could apply the treatment into clinical practice to improve the cognitive deficits for patients with mild AD.

## Supporting Information

S1 CONSORT Checklist (DOC)

S1 CONSORT Extension Extension for Herbal Interventions. (PDF)

S1 Fig Approved file of Ethical Committee. The trial was approved by the Ethics Committee of the Second Affiliated Hospital of Tianjin University of TCM in July 26, 2011.
(TIF)

S2 Fig The main chemicals in the decoction of YHD. (TIF)

S1 Methods and Materials Quality control of the herbal concentrate-granules, Procedure of herbal concentrate-granules, and Direction of herbal concentrate-granules intake. (DOC)

S1 Table YHD formula of each dose. (DOCX)

S2 Table Lot number of Chinese herbs, DH, and the simulations. (DOCX)

S1 Trial Protocol (Chinese). (DOCX)

S2 Trial Protocol (English). (DOCX)
