# Correction: Cognitive Improvement during Treatment for Mild Alzheimer's Disease with a Chinese Herbal Formula: A Randomized Controlled Trial

**DOI:** 10.1371/journal.pone.0199895

**Published:** 2018-06-25

**Authors:** Yulian Zhang, Cuiru Lin, Linlin Zhang, Yuanwu Cui, Yun Gu, Jiakui Guo, Di Wu, Qiang Li, Wanshan Song

The affiliation for the third author is incorrect. Linlin Zhang is not affiliated with #2 but with #1 Department of Acupuncture and Cerebropathy, Second Affiliated Hospital of Tianjin University of Traditional Chinese Medicine, Tianjin, China.
