# Supplementary material for: Cognitive Improvement during Treatment for Mild Alzheimer’s Disease with a Chinese Herbal Formula: A Randomized Controlled Trial
Source: PLoS One. 2015 Jun 15;10(6):e0130353. doi: 10.1371/journal.pone.0130353 (PMC4468068; doi:10.1371/journal.pone.0130353)
Supplement: S1 CONSORT Extension — (PDF) [file pone.0130353.s002.pdf]

Annals of Internal Medicine | Academia and Clinic

Reporting Randomized, Controlled Trials of Herbal Interventions: An Elaborated CONSORT Statement

Joel J. Gagnier, ND, MSc; Heather Boon, PhD; Paula Rochon, MD, MPH; David Moher, PhD; Joanne Barnes, PhD, MRPharmS FLS; and Claire Bombardier, MD, for the CONSORT Group*

Herbal medicinal products are widely used, vary greatly in content and quality, and are actively tested in randomized, controlled trials (RCTs). The authors' objective was to develop recommendations for reporting RCTs of herbal medicine interventions, based on the need to elaborate on the 22-item CONSORT (Consolidated Standards of Reporting Trials) checklist. Telephone calls were made and a consensus meeting was held with 16 participants in Toronto, Canada, to develop these recommendations. The group agreed on context-specific elaborations of 9 CONSORT checklist items for RCTs of herbal medicines. Item 4, concerning the herbal medicine intervention, required the most extensive elaboration. These recommendations have been developed to improve the reporting of RCTs using herbal medicine interventions.

Ann Intern Med. 2006;144:364-367. www.annals.org
For author affiliations, see end of text.
*The members of the CONSORT Group are listed on the following Web site: www.consort-statement.org/profiles/partners.html.

Randomized, controlled trials (RCTs) of herbal interventions often inadequately describe important aspects of their methods (1–4). Although the quality of reporting of these trials may be improving with time, many still lack important information, particularly about the composition of the herbal intervention (4, 5). Crude herbal drugs are natural products and their chemical composition varies depending on several factors, such as geographic source of the plant material, climate in which it was grown, and time of harvest. Commercially available herbal medicinal products also vary in their content and concentration of chemical constituents from batch to batch and when products containing the same herbal ingredient are compared among manufacturers (6–14). Even when herbal products are standardized for content of known active or marker compounds to achieve more consistent pharmaceutical quality, there is variation in the concentrations of other constituents. These variations can result in differences in pharmacologic activity in vitro (15) and in bioavailability in humans (16). Mindful of these issues, we elaborated on the 22-item checklist of the Consolidated Standards of Reporting Trials (CONSORT) statement (17) to help authors and editors improve reporting of RCTs of herbal interventions.

Methods

We developed these reporting recommendations in 3 phases that included premeeting item generation, a consensus meeting, and postmeeting feedback. The individuals who participated are listed in the Appendix (available at www.annals.org). To generate items, 1 investigator conducted telephone interviews of 16 participants with expertise in the method and reporting of RCTs (5 participants), pharmacognosy (4 participants), herbal medicinal products (5 participants), medical statistics (1 participant), and herbal product manufacturing (1 participant). The investigator asked participants to suggest revisions to existing CONSORT checklist items and also to additional items required for reporting trials of herbal interventions. He asked participants to nominate revisions or new items on the basis of empirical evidence that not reporting the item would bias estimates of treatment effect. When no empirical evidence was available, commonsense reasoning was acceptable. After completing all telephone calls, the investigator thematically grouped items and circulated them by e-mail to each participant for review.

Fourteen participants attended the consensus meeting. The meeting began with a review of the premeeting checklist item suggestions. We emphasized minimizing item elaborations and additions and basing elaborations on evidence whenever possible. Each item suggestion was presented and followed by debate for its inclusion, deletion, or modification. This process was repeated until all items were reviewed and a consensus emerged.

After the consensus meeting, we circulated a draft summary report to all participants to ensure that it accurately represented decisions made during the consensus meeting. We then circulated the report to the wider CONSORT Group for input and revised it on the basis of their suggestions. Ethical approval was obtained from The University of Toronto Health Sciences Ethics Review Committee on 23 January 2004.

Financial support for the consensus meeting was provided by the Canadian Institutes of Health Research. The funding body had no role in the design, conduct, or analysis of this study and did not influence the decision to submit the manuscript for publication. All researchers are independent of the funders.

See also:

Web-Only
Appendix
Appendix Table
Conversion of figure and tables into slides

 © 2006 American College of Physicians

Table. **Proposed Elaboration of CONSORT Checklist Item 4 for Reporting Randomized, Controlled Trials of Herbal Medicine Interventions***

Paper Section and Topic	Item	Descriptor	Examples of Good Reporting†
Methods			
Interventions	4	Where applicable, the description of an herbal intervention should include:	
	4A: Herbal medicinal product name	1. The Latin binomial name together with botanical authority and family name for each herbal ingredient; common name(s) should also be included.	The herbal medicine intervention used in this trial was an extract of *Ginkgo biloba L.* (Ginkgoaceae; maidenhair tree).
		2. The proprietary product name (i.e., brand name) or the extract name (e.g., EGb-761) and the name of the manufacturer of the product.	The product used was LI 1370, an extract of *Ginkgo biloba L.*, manufactured by Lichtwer Pharma (Berlin, Germany) (18).
		3. Whether the product used is authorized (licensed, registered) in the country in which the study was conducted.	This product is registered for use as a natural health product in Canada.
	4B: Characteristics of the herbal product	1. The part(s) of plant used to produce the product or extract.	The extract was obtained from leaves of *Ginkgo biloba L.*
		2. The type of product used (e.g., raw [fresh or dry], extract).	The herbal medicine intervention was an extract of *Ginkgo biloba L.*
		3. The type and concentration of extraction solvent used (e.g., 80% ethanol, 100% H_2O, 90% glycerine, etc.) and the ratio of herbal drug to extract (e.g., 2 to 1).	The solvent used in the extract was alcohol (80% ethanol) and the ratio of herbal drug to extract was 5 to 1.
		4. The method of authentication of raw material (i.e., how done and by whom) and the lot number of the raw material. State if a voucher specimen (i.e., retention sample) was retained and, if so, where it is kept or deposited, and the reference number.	A staff botanist visually identified the growing plant. The lot number for the *Ginkgo biloba L.* extract used in this study was #557-05. A voucher specimen was retained (#23-673) and is kept at the manufacturer headquarters in Toronto, Canada.
	4C: Dosage regimen and quantitative description	1. The dosage of the product, the duration of administration, and how these were determined.	Each capsule contained 60 mg of the extract. A total of 3 capsules were given each day, 1 before each of 3 meals, for 3 months. This dosage regimen was determined by referring to previous clinical trials testing the effects of similar *Ginkgo biloba L.* extracts for the same indication.
		2. The content (e.g., as weight, concentration; may be given as range where appropriate) of all quantified herbal product constituents, both native and added, per dosage unit form. Added materials, such as binders, fillers, and other excipients (e.g., 17% maltodextrin, 3% silicon dioxide per capsule), should also be listed.	The percentages of quantified chemical constituents per capsule was as follows: 15 mg (25%) flavonoids, 3 mg (5%) ginkgolides, 1.8 mg (3%) bilobalides.
		3. For standardized products, the quantity of active/marker constituents per dosage unit form.	The percentages of marker constituents per capsule were as follows: 25% flavonoids, 5% ginkgolides, 3% bilobalides.
	4D: Qualitative testing	1. Product's chemical fingerprint and methods used (equipment and chemical reference standards) and who performed the chemical analysis (e.g., the name of the laboratory used); whether a sample of the product (i.e., retention sample) was retained and if so, where it is kept or deposited.	The high-pressure liquid chromatography chemical fingerprint for the extract of *Ginkgo biloba L.* can be seen in the **Figure** (19). The method for performing this analysis was as follows: High-pressure liquid chromatography was achieved using a minibore Phenomenex Luna 5-μm C_{18} (2) column with dimensions 250 × 2.00 mm at 45 °C with a one-step linear gradient using acetonitrile:formic acid (0.3%) at a flow rate of 0.4 mL/min (20). The analysis was done by an individual with 12 years' experience in the methods, at an independent laboratory, CanHerba Labs Inc. (Windsor, Ontario, Canada). The product sample is also kept at CanHerba Labs Inc.
		2. Description of any special testing/purity testing (e.g., heavy metal or other contaminant testing) undertaken, which unwanted components were removed and how (i.e., methods).	Laboratory personnel were blinded to the identity of the extract and control capsules. Concentrations (μg/g) of lead, mercury, and arsenic were measured by x-ray fluorescence spectroscopy 23 equipped with a tungsten x-ray tube, a Si(Li)-semiconductor detector, and software version 2.2R03 I (Spectro Analytical Instruments, Kleve, Germany). National Institute of Standards and Technology solid standard reference materials 2709, 2710, 2711, 24, and liquid certified standards (SCP Science, Champlain, New York) containing specified heavy metal concentrations served as positive and negative controls (21).

Continued on following page

Table—**Continued**

Paper Section and Topic	Item	Descriptor	Examples of Good Reporting
		3. Standardization: what to standardize (e.g., which chemical components of the product) and how (e.g., chemical processes or biological/functional measures of activity).	The *Ginkgo biloba L.* extract used in this trial was standardized to contain 25% flavonoids, 5% ginkgolides, and 3% bilobalides. Methods included high-pressure liquid chromatography using a minibore Phenomenex Luna 5-μm C_{18} (2) column with dimensions 250 × 2.00 mm at 45 °C with a one-step linear gradient using acetonitrile:formic acid (0.3%) at a flow rate of 0.4 mL/min (3). We used the following reference standards: bilobalide (95%), ginkgolides A (90%), B (95%), C (95%), J (99%) purchased from HerbalChems (San Francisco, California)‡, and Quercetin (95%) purchased from Sigma (St. Louis, Missouri) and kaempferol (90%) and isorhamnetin (99%) purchased from Indofine Chemical Company (Hillsborough, New Jersey). The purity of these reference standards was assumed as provided by the suppliers (3).
	4E: Placebo/control group	The rationale for the type of control/placebo used.	The placebo capsules used in this trial were identically sized capsules filled with lactose powder, and colored (with food coloring) to match the *Ginkgo biloba L.* capsules.
	4F: Practitioner	A description of the practitioners (e.g., training and practice experience) that are a part of the intervention.	Clinicians choosing the appropriate treatment and dosage were trained as primary care physicians; were licensed in Ontario, Canada; had been practicing medicine for an average of 12 years; and had attended continuing medical education lectures on evidence-based herbal medicine interventions.

* CONSORT = Consolidated Standards of Reporting Trials.
† Examples included are not from actual publications unless directly referenced. They were developed explicitly to provide extremely specific and concise examples of good reporting for each item. All examples are for the same herbal medicine intervention, which contains just 1 herbal medicinal product, *Ginkgo biloba L.* Referenced sections were changed slightly from the original reports to be consistent with respect to the particular herbal medicine intervention used across these examples.
‡ This is a fictional company that was added for the completeness of the report.

Results

The group did not recommend any new CONSORT checklist items or modifications in the CONSORT flow diagram. We did, however, elaborate on 9 of the 22 CONSORT checklist items to enhance their relevance to trials of herbal interventions (**Table**, **Figure**; **Appendix Table**, available at www.annals.org), including minor recommendations for 8 items (item 1 [title and abstract], item 2 [background], item 3 [participants], item 6 [outcomes], item 15 [baseline data], item 20 [interpretation], item 21 [generalizability], and item 22 [overall evidence]) and detailed recommendations for 1 item (item 4 [interventions]).

Figure. **The high-pressure liquid chromatography chemical fingerprint for the extract of *Ginkgo biloba L.* (19)**

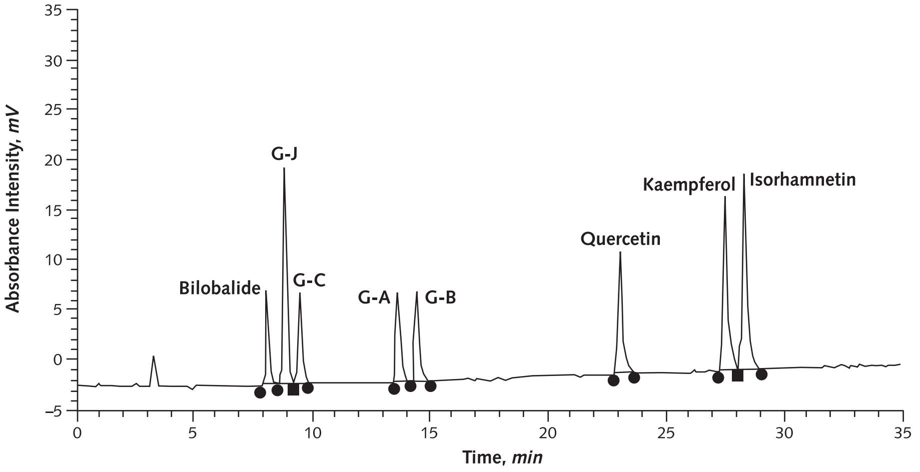

G-A = ginkgolide A; G-B = ginkgolide B; G-C = ginkgolide C; G-J = ginkgolide J.

The **Table** shows the detailed recommendations for item 4 and an example of good reporting related to each recommendation. These recommendations begin with the words "where applicable" to indicate that all information suggested may not be applicable to every type of herbal medicine intervention. For example, an herbal medicinal product comprising crude herbal material (for example, leaves and stems) simply prepared as a tea or decoction does not require description of the "type and concentration of solvent used and the ratio of herbal drug to extract" (item 4B.3). Also, not every herbal medicine intervention will have a finished product or extract name or manufacturer (item 4A.2), but instead may be made by the investigators specifically for the study. In such circumstances, all methods used in preparing and formulating the product must be reported. Similarly, item 4F is not required for herbal interventions when the practitioner is not a part of the intervention. With these exceptions, we recommend that all information shown in the **Table** be reported for all herbal interventions.

Discussion

We developed recommendations to be used in conjunction with the existing CONSORT checklist when reporting RCTs of herbal interventions. In particular, we thought it imperative that reports of RCTs provide clear and complete descriptions of the herbal intervention. We think that our recommendations might also be relevant for reporting herbal interventions in other research designs, whether preclinical (for example, in vivo or in vitro) or clinical (for example, N of 1 trials), and refer interested readers to a detailed explanatory document that further describes each of our recommendations and provides additional examples of good reporting (22). We hope that authors find our recommendations instructive and that journals will endorse their use and modify their instructions to authors accordingly.

From University of Toronto, Institute for Evaluative Sciences, Institute for Work and Health, and Toronto General Research Institute, Toronto, Canada; Children's Hospital of Eastern Ontario, Ottawa, Canada; and University of London, London, United Kingdom.

Acknowledgments: The authors thank Greer Palloo for aiding in the preparation for the June meeting, and Jaime DeMelo, ND, and Cyndi Gilbert for assisting Joel Gagnier, ND, MSc, and Claire Bombardier, MD, during the actual meeting procedures.

Grant Support: This study was funded in part by an operating grant from the Canadian Institutes of Health Research, Clinical Trials Divisions (ATF-66679). Dr. Gagnier is supported by a postgraduate fellowship from the Canadian Institutes of Health Research and the Natural Health Products Directorate.

Potential Financial Conflicts of Interest: *Grants received:* J. Barnes (Lichtwer Pharma); *Grants pending:* J. Barnes (Lichtwer Pharma); *Royalties:* J. Barnes (Pharmaceutical Press, Churchill Livingstone).

Requests for Single Reprints: Joel J. Gagnier, ND, MSc, 5955 Ontario Street, Unit 307, Windsor, Ontario N81S W6, Canada; e-mail, j.gagnier@utoronto.ca.

Current author addresses are available at www.annals.org.

References

1. **Little C, Parsons T.** Herbal therapy for treating rheumatoid arthritis. Cochrane Library. 2000;4.
2. **Linde K, Jonas WB, Melchart D, Willich S.** The methodological quality of randomized controlled trials of homeopathy, herbal medicines and acupuncture. Int J Epidemiol. 2001;30:526-31. [PMID 11416076]
3. **Moher D, Sampson M, Campbell K, Beckner W, Lepage L, Gaboury I, et al.** Assessing the quality of reports of randomized trials in pediatric and complementary and alternative medicine. BMC Pediatr. 2002;2:1-6.3.
4. **Klassen TP, Pham B, Lawson ML, Moher D.** For randomized controlled trials, the quality of reports of complementary and alternative medicine was as good as reports of conventional medicine. J Clin Epidemiol. 2005;58:763-8. [PMID: 16018911]
5. **Wolsko PM, Solondz DK, Phillips RS, Schachter SC, Eisenberg DM.** Lack of herbal supplement characterization in published randomized controlled trials. Am J Med. 2005;118:1087-93. [PMID: 16194636]
6. **Harkey MR, Henderson GL, Gershwin ME, Stern JS, Hackman RM.** Variability in commercial Ginseng products: an analysis of 25 preparations. Am J Clin Nutr. 2002;75:600-1. [PMID: 11382666]
7. **Groenewegen WA, Heptinstall S.** Amounts of feverfew in commercial preparations of the herb [Letter]. Lancet. 1986;1:44-5. [PMID: 2867286]
8. **Heptinstall S, Awang DV, Dawson BA, Kindack D, Knight DW, May J.** Parthenolide content and bioactivity of feverfew (*Tanacetum parthenium* (*L.*) Schultz-Bip.). Estimation of commercial and authenticated feverfew products. J Pharm Pharmacol. 1992;44:391-5. [PMID: 1359053]
9. **Nelson MH, Cobb SE, Shelton J.** Variations in parthenolide content and daily dose of feverfew products. Am J Health Syst Pharm. 2002;59:1527-31. [PMID: 12185827]
10. **Manning J, Roberts JC.** Analysis of catechin content of commercial green tea products. J Herb Pharmacother. 2003;3:19-32. [PMID: 15277054]
11. **Carlson M, Thompson RD.** Liquid chromatographic determination of methylxanthines and catechins in herbal preparations containing guaraná. J AOAC Int. 1998;81:691-701. [PMID: 9680692]
12. **Haller CA, Duan M, Benowitz NL, Jacob P 3rd.** Concentrations of ephedra alkaloids and caffeine in commercial dietary supplements. J Anal Toxicol. 2004; 28:145-51. [PMID: 15107143]
13. **Kressmann S, Müller WE, Blume HH.** Pharmaceutical quality of different *Ginkgo biloba* brands. J Pharm Pharmacol. 2002;54:661-9. [PMID: 12005361]
14. **Gurley BJ, Gardner SF, Hubbard MA.** Content versus label claims in ephedra-containing dietary supplements. Am J Health Syst Pharm. 2000;57:963-9. [PMID: 10832496]
15. **Schulte-Löbbert S, Holoubek G, Müller WE, Schubert-Zsilavecz M, Wurglics M.** Comparison of the synaptosomal uptake inhibition of serotonin by St John's wort products. J Pharm Pharmacol. 2004;56:813-8. [PMID: 15231048]
16. **Kressmann S, Biber A, Wonnemann M, Schug B, Blume HH, Müller WE.** Influence of pharmaceutical quality on the bioavailability of active components from *Ginkgo biloba* preparations. J Pharm Pharmacol. 2002;54:1507-14. [PMID: 12495553]
17. **Moher D, Schulz KF, Altman DG.** The CONSORT statement: revised recommendations for improving the quality of reports of parallel-group randomized trials. Ann Intern Med. 2001;134:657-62. [PMID: 11304106]
18. **Drew S, Davies E.** Effectiveness of *Ginkgo biloba* in treating tinnitus: double blind, placebo controlled trial. BMJ. 2001;321:1-6. [PMID: 11154618]
19. **Li W, Fitzloff JF.** Simultaneous determination of terpene lactones and flavonoid aglycones in *Ginkgo biloba* by high-performance liquid chromatography with evaporative light scattering detection. J Pharm Biomed Anal. 2002;30:67-75. [PMID: 12151066]
20. **Dubber MJ, Kanfer I.** High-performance liquid chromatographic determination of selected flavonols in *Ginkgo biloba* solid oral dosage forms. J Pharm Pharm Sci. 2004;7:303-9. [PMID: 15576009]
21. **Saper RB, Kales SN, Paquin J, Burns MJ, Eisenberg DM, Davis RB, et al.** Heavy metal content of ayurvedic herbal medicine products. JAMA. 2004;292: 2868-73. [PMID: 15598918]
22. **Gagnier JJ, Boon H, Rochon P, Barnes, J, Moher D, Bombardier CB, for the CONSORT Group.** Recommendations for reporting randomized controlled trials of herbal interventions: explanation and elaboration. J Clin Epidemiol. 2006 [In press].

Current Author Addresses: Dr. Gagnier: 5955 Ontario Street, Unit 307, Windsor, Ontario N81S W6, Canada.
Dr. Boon: University of Toronto, Faculty of Pharmacy, 19 Russell Street, Toronto, Ontario M5S 2S2, Canada.
Dr. Rochon: Baycrest Centre, 3560 Bathurst Street, Toronto, Ontario M6A 2E1, Canada.
Dr. Moher: Children's Hospital of Eastern Ontario Research Institute, 401 Smyth Road, Room 210, Ottawa, Ontario K1H 8L1, Canada.
Dr. Barnes: Centre for Pharmacognosy and Phytotherapy, University of London School of Pharmacy, 29/39 Brunswick Square, London WC1N 1AX, United Kingdom.
Dr. Bombardier: Institute for Work and Health, 481 University Avenue, Suite 800, Toronto, Ontario M5G 2E9, Canada.

Appendix

The following individuals participated in the premeeting telephone calls or attended the consensus meeting and provided input toward the elaborations to existing CONSORT checklist items: Doug Altman (Cancer Research UK Medical Statistics Group, Centre for Statistics in Medicine, Oxford, United Kingdom); Joanne Barnes (Centre for Pharmacognosy and Phytotherapy, The School of Pharmacy, University of London, London, United Kingdom); Claire Bombardier, *Meeting Chair* (Department of Health Policy Management and Evaluation, Faculty of Medicine, University of Toronto, Toronto, Ontario, Canada); Heather Boon (Leslie Dan Faculty of Pharmacy, University of Toronto, Toronto, Canada); Mark Blumenthal (American Botanical Council, Austin, Texas); Ranjit Roy Chaudhury, *Chair* (International Clinical Epidemiology Network [INCLEN Inc.], New Delhi, India); Philip Devereaux (Department of Clinical Epidemiology and Biostatistics, Faculty of Health Sciences, McMaster University, Hamilton, Ontario, Canada); Theo Dingermann (Institut for Pharmaceutical Biology Biozentrum, University of Frankfurt/Main, Frankfurt am Main, Germany); Joel Gagnier, *Meeting Coordinator* (Department of Health Policy Management and Evaluation, Faculty of Medicine, University of Toronto, Toronto, Ontario, Canada); Gary Leong (Jamieson Vitamins Inc., Windsor, Ontario, Canada); Allison McCutcheon (Faculty of Pharmaceutical Sciences, University of British Columbia, Vancouver, British Columbia, Canada); David Moher (Children's Hospital of Eastern Ontario Research Institute, Ottawa, Ontario, Canada); Max H. Pittler (Complementary Medicine, Peninsula Medical School, University of Exeter, Exeter, United Kingdom); David Riley (University of New Mexico Medical School, Santa Fe, New Mexico); Paula Rochon (Baycrest Centre, Toronto, Ontario, Canada); Michael Smith (Health Canada, Natural Health Products Directorate, Ottawa, Ontario, Canada); and Andrew Vickers (Memorial Sloan-Kettering Cancer Center, New York, New York).

Appendix Table. **Proposed Elaborations of CONSORT Items for Randomized, Controlled Trials of Herbal Medicine Interventions***

Paper Section and Topic	Item Number	Descriptor	Reported on Page Number
**Title and Abstract**	1	How participants were allocated to interventions (e.g., "random allocation," "randomized" or "randomly assigned").<br>*Either the title or abstract, or both, should state the herbal medicinal product's Latin binomial, the part of the plant used, and the type of preparation.*	1-2
**Introduction**			
Background	2	Scientific background and explanation of rationale.<br>*Including a brief statement of reasons for the trial with reference to the specific herbal medicinal product being tested and, if applicable, whether new or traditional indications are being investigated.*	3-4
**Methods**			
Participants	3	Eligibility criteria for participants and the settings and locations where the data were collected.<br>*If a traditional indication is being tested, a description of how the traditional theories and concepts were maintained. For example, participant inclusion criteria should reflect the theories and concepts underlying the traditional indication.*	5-6
Interventions	4	Precise details of the interventions intended for each group and how and when they were actually administered.	
	4A: Herbal medicinal product name	*1. The Latin binomial name and the botanical authority and family name for each herbal ingredient; common name(s) should also be included.*<br>*2. The proprietary product name (i.e., brand name) or the extract name (e.g., EGb-761) and the name of the manufacturer of the product.*<br>*3. Whether the product used is authorized (licensed, registered) in the country in which the study was conducted.*	6-7
	4B: Characteristics of the herbal product	*1. The part(s) of plant used to produce the product or extract.*<br>*2. The type of product used (e.g., raw [fresh or dry], extract).*<br>*3. The type and concentration of extraction solvent used (e.g., 80% ethanol, 100% H_2O, 90% glycerine, etc.) and the ratio of herbal drug to extract (e.g., 2 to 1).*<br>*4. The method of authentication of raw material (i.e., how done and by whom) and the lot number of the raw material. State if a voucher specimen (i.e., retention sample) was retained and, if so, where it is kept or deposited, and the reference number.*	6-7
	4C: Dosage regimen and quantitative description	*1. The dosage of the product, the duration of administration, and how these were determined.*<br>*2. The content (e.g., as weight, concentration; may be given as range where appropriate) of all quantified herbal product constituents, both native and added, per dosage unit form. Added materials, such as binders, fillers, and other excipients (e.g., 17% maltodextrin, 3% silicon dioxide per capsule), should also be listed).*<br>*3. For standardized products, the quantity of active/marker constituents per dosage unit form.*	6-7
	4D: Qualitative testing	*1. Product's chemical fingerprint and methods used (equipment and chemical reference standards) and who performed the chemical analysis (e.g., the name of the laboratory used). Whether a sample of the product (i.e., retention sample) was retained and if so, where it is kept or deposited.*<br>*2. Description of any special testing/purity testing (e.g., heavy metal or other contaminant testing) undertaken; which unwanted components were removed and how (i.e., methods).*<br>*3. Standardization: what to standardize (e.g., which chemical components of the product) and how (e.g., chemical processes, or biological/functional measures of activity).*	7
	4E: Placebo/control group	*The rationale for the type of control or placebo used.*	7
	4F: Practitioner	*A description of the practitioners (e.g., training and practice experience) who are a part of the intervention.*	7
Objectives	5	Specific objectives and hypotheses.	4
Outcomes	6	Clearly defined primary and secondary outcome measures and, when applicable, any methods used to enhance the quality of measurements (e.g., multiple observations, training of assessors).<br>*Outcome measures should reflect the intervention and indications tested considering, where applicable, underlying theories and concepts.*	7-8
Sample size	7	How sample size was determined and, when applicable, explanation of any interim analyses and stopping rules.	8
Randomization			
Sequence allocation	8	Method used to generate the random allocation sequence, including details of any restriction (e.g., blocking, stratification).	6
Allocation concealment	9	Method used to implement the random allocation sequence (e.g., numbered containers or central telephone), clarifying whether the sequence was concealed until interventions were assigned.	6
Implementation	10	Who generated the allocation sequence, who enrolled participants, and who assigned participants to their groups.	6

Continued on following page

Appendix Table—Continued

Paper Section and Topic	Item Number	Descriptor	Reported on Page Number
Blinding (masking)	11	Whether or not participants, those administering the interventions, and those assessing the outcomes were blinded to group assignment. If done, how the success of blinding was evaluated.	6
Statistical methods	12	Statistical methods used to compare groups for primary outcome(s); methods for additional analyses, such as subgroup analyses and adjusted analyses.	8-9
**Results**			
Participant flow	13	Flow of participants through each stage (a diagram is strongly recommended). Specifically, for each group report the numbers of participants randomly assigned, receiving intended treatment, completing the study protocol, and analyzed for the primary outcome. Describe protocol deviations from study as planned, together with reasons.	9
Recruitment	14	Dates defining the periods of recruitment and follow-up.	9
Baseline data	15	Baseline demographic and clinical characteristics of each group. *Including concomitant medications, herbal and complementary medicine use.*	10-11
Numbers analyzed	16	Number of participants (denominator) in each group included in each analysis and whether the analysis was by "intention-to-treat." State the results in absolute numbers when feasible (e.g., 10/20, not 50%).	8-9
Outcomes and estimation	17	For each primary and secondary outcome, a summary of results for each group, and the estimated effect size and its precision (e.g., 95% confidence interval).	11-13
Ancillary analyses	18	Address multiplicity by reporting any other analyses performed, including subgroup analyses and adjusted analyses, indicating those pre-specified and those exploratory.	12
Adverse events	19	All important adverse events or side effects in each intervention group.	13
**Discussion**			
Interpretation	20	Interpretation of the results, taking into account study hypotheses, sources of potential bias or imprecision, and the dangers associated with multiplicity of analyses and outcomes. *Interpretation of the results in light of the product and dosage regimen used.*	14-15
Generalizability	21	Generalizability (external validity) of the trial findings. *Where possible, discuss how the herbal product and dosage regimen used relate to what is used in self-care and/or practice.*	15
Overall evidence	22	General interpretation of the results in the context of current evidence. *Discussion of the trial results in relation to trials of other available products.*	15

* CONSORT items (1, 17) are listed in normal text. Proposed recommendations for reports of herbal medicine and randomized, controlled trials are listed in italicized text. CONSORT = Consolidated Standards of Reporting Trials.
