# Supplementary figures and images for: Cognitive Improvement during Treatment for Mild Alzheimer’s Disease with a Chinese Herbal Formula: A Randomized Controlled Trial
Source: PLoS One. 2015 Jun 15;10(6):e0130353. doi: 10.1371/journal.pone.0130353 (PMC4468068; doi:10.1371/journal.pone.0130353)

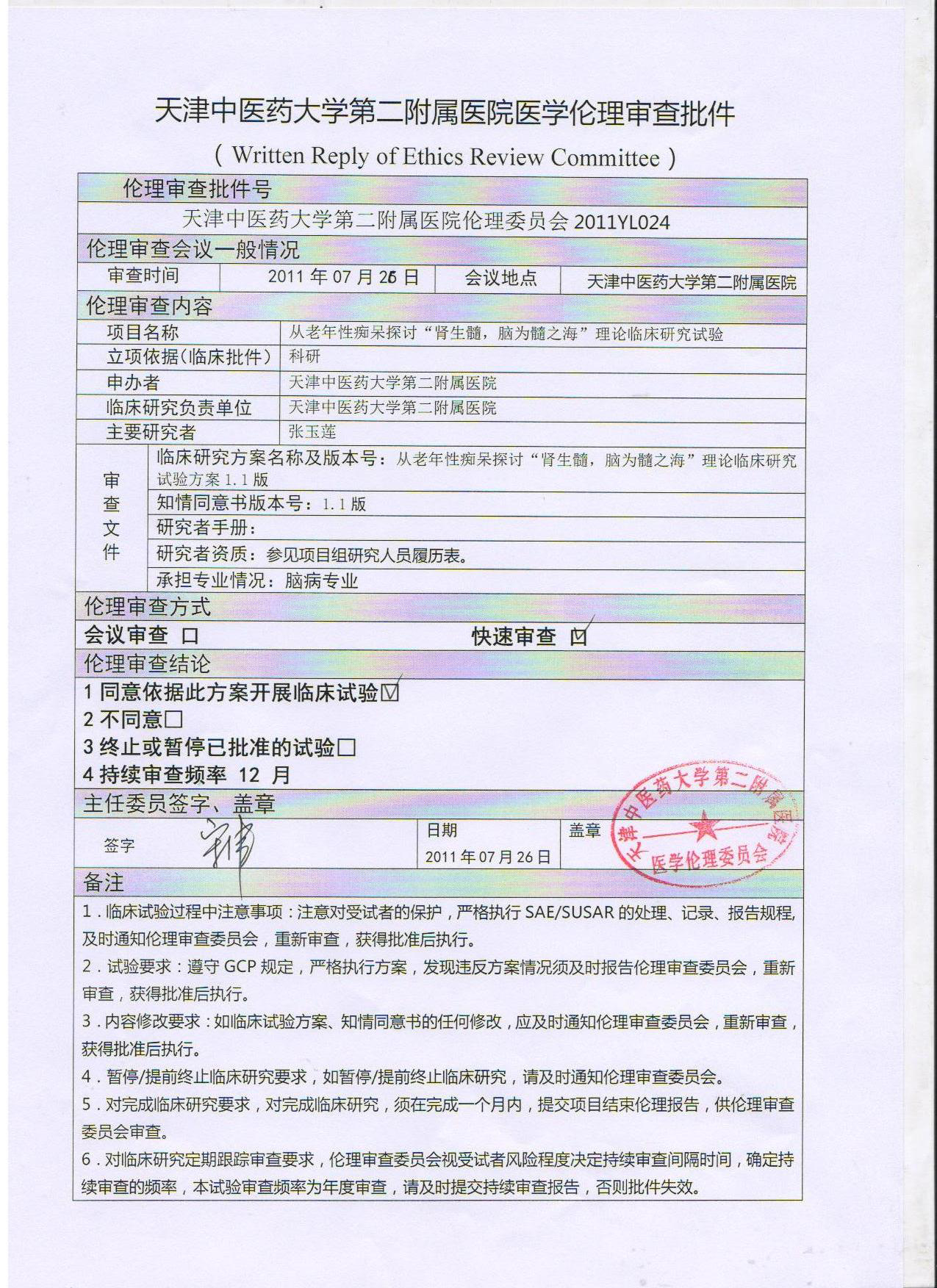

Supplement: S1 Fig — The trial was approved by the Ethics Committee of the Second Affiliated Hospital of Tianjin University of TCM in July 26, 2011. (TIF) [file pone.0130353.s003.tif]

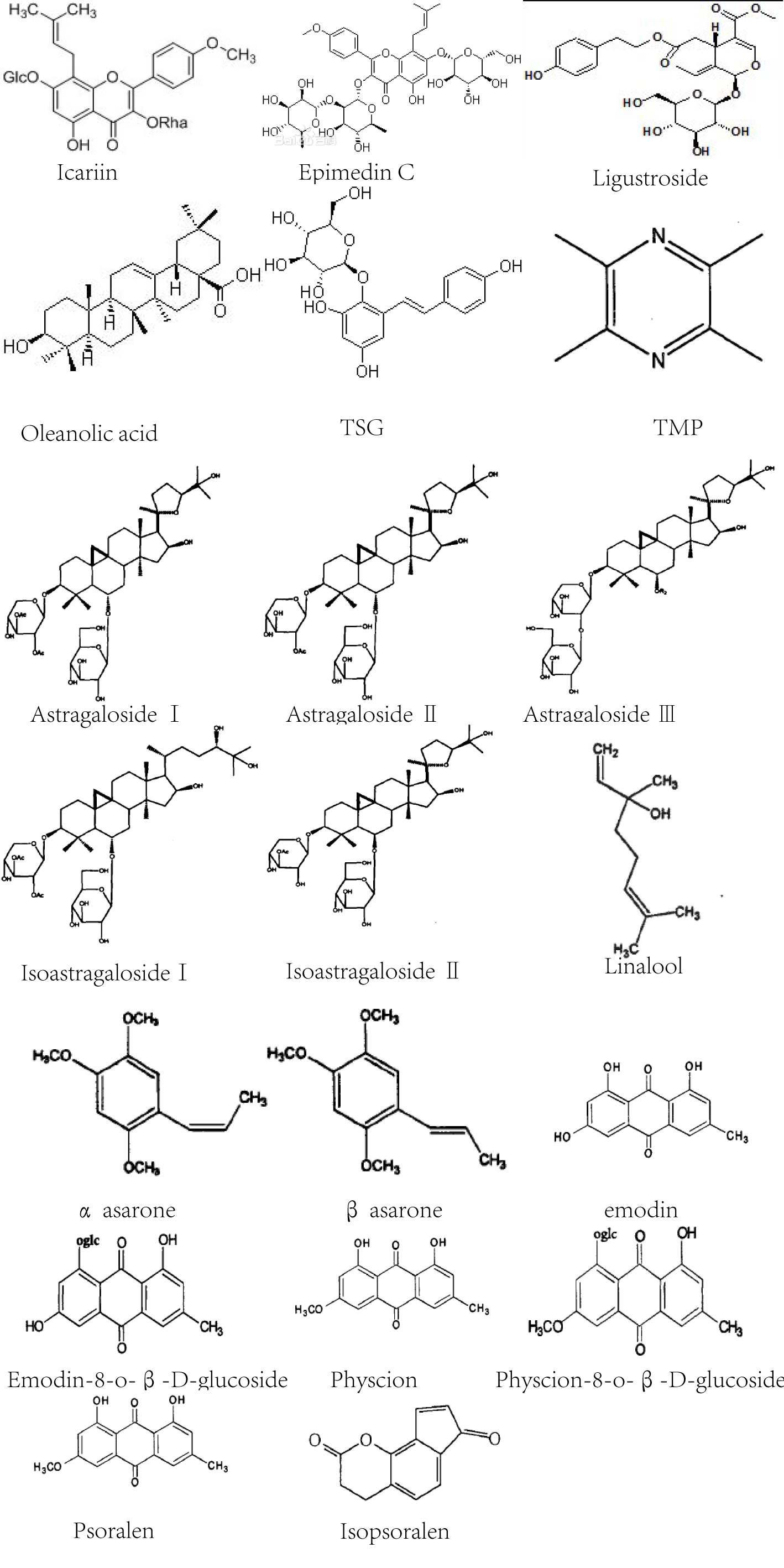

Supplement: S2 Fig — (TIF) [file pone.0130353.s004.tif]
