# Supplementary material for: Cognitive Improvement during Treatment for Mild Alzheimer’s Disease with a Chinese Herbal Formula: A Randomized Controlled Trial
Source: PLoS One. 2015 Jun 15;10(6):e0130353. doi: 10.1371/journal.pone.0130353 (PMC4468068; doi:10.1371/journal.pone.0130353)
Supplement: S1 Methods and Materials — (DOC) [file pone.0130353.s005.doc]

**Supporting Methods and Materials**

**Quality control of the herbal concentrate-granules**

We use herbal concentrate-granules in this trial. All granules used in this study were from the same place and in the same batch. According to the determination method recorded in the “China Pharmacopoeia (2010 edition)”, the herbs were conformed to meet the China Pharmacopoeia standard (Committee, 2010).

**Procedure of herbal concentrate-granules**

Firstly, put one herb in a certain dosage into the boiling pot, and add water and soak for 1 hour. Secondly, boil the herb for 50 minutes and filtrate the decoction. Thirdly, concentrate the decoction and dry it. Fourthly, add excipients and pelletize into granules. Fifthly, package the granules in a bag. (Due to intellectual property and secret, the procedure was provided briefly by Shenzhen Sanjiu Modern Chinese Medicine limited Company)

**Direction of herbal concentrate-granules intake**

Firstly, put half bag granules of each herb in the formula into a cup and add 100ml boiled water. Secondly, stir the decoction to solve the granules. Thirdly, drink the decoction until it is suitable.
