# Supplementary material for: Cognitive Improvement during Treatment for Mild Alzheimer’s Disease with a Chinese Herbal Formula: A Randomized Controlled Trial
Source: PLoS One. 2015 Jun 15;10(6):e0130353. doi: 10.1371/journal.pone.0130353 (PMC4468068; doi:10.1371/journal.pone.0130353)
Supplement: S1 Table — (DOCX) [file pone.0130353.s006.docx]

S1 Table YHD formula of each dose.

| **Herbal Ingredients** | **YHD** |
| --- | --- |
| Epimedium **(g)** | 10 |
| Psoralea fruit **(g)** | 10 |
| Radix Polygoni Multiflori **(g)** | 10 |
| Radix Astragali **(g)** | 10 |
| Ligusticum wallichi Franchat **(g)** | 6 |
| Fructus Ligustri Lucidi**(g)** | 10 |
| Acorus Gramineus**(g)** | 6 |
| **Total Weight (g)** | 62 |
|  |  |
