# Supplementary material for: Cognitive Improvement during Treatment for Mild Alzheimer’s Disease with a Chinese Herbal Formula: A Randomized Controlled Trial
Source: PLoS One. 2015 Jun 15;10(6):e0130353. doi: 10.1371/journal.pone.0130353 (PMC4468068; doi:10.1371/journal.pone.0130353)
Supplement: S2 Table — (DOCX) [file pone.0130353.s007.docx]

**S2 Table** Lot number of Chinese herbs, DH, and the simulations**.**

|  | Drug | Simulation | Manufacturer |
| --- | --- | --- | --- |
| Epimedium | 1112001S | 1111001S | Shenzhen Sanjiu Modern Chinese Medicine limited Company (Tianjin, China) |
| Psoralea fruit | 1103291S | 1111001S |  |
| Radix Polygoni Multiflori | 1011032S | 1111001S |  |
| Radix Astragali | 1103041S | 1111001S |  |
| Ligusticum Chuanxiong | 1103141S | 1111001S |  |
| Fructus Ligustri Lucidi | 1011211S | 1111001S |  |
| Acorus Gramineus | 1103251S | 1111001S |  |
| Donepezil hydrochloride | 110507A | 110501B | Eisai China Inc. (Tianjin, China) |
