# Supplementary material for: Cognitive Improvement during Treatment for Mild Alzheimer’s Disease with a Chinese Herbal Formula: A Randomized Controlled Trial
Source: PLoS One. 2015 Jun 15;10(6):e0130353. doi: 10.1371/journal.pone.0130353 (PMC4468068; doi:10.1371/journal.pone.0130353)
Supplement: S1 Trial Protocol — (DOCX) [file pone.0130353.s008.docx]

**实验方案**

**从老年性痴呆探讨“肾生髓，脑为髓之海”理论的研究**

**国家重点基础研究发展计划（973计划）**( 2010CB530405).

**ChiCTR-TRC-12002846**

**经费来源：**国家科技部

**研究疾病：** 老年痴呆

**研究类型：**干预性研究

**研究设计:** 随即平行对照

**负责单位：**天津中医药大学第二附属医院

**负责人：** 张玉莲

**方案拟定时间：**2010-1-1

**研究实施时间：**2010-1-1 至 2014-12-31

**研究实施单位：**天津中医药大学第二附属医院

上海中医药大学附属龙华医院

天津市环湖医院

**主要研究者：**张玉莲

天津市河北区真理道816号

Tel. +86-13821151196

Email: zhyl220@126.com

目录

[1.研究内容 2](#_Toc409529957)

[1.1补肾填精法治疗老年性痴呆临床疗效评价研究 2](#_Toc409529958)

[1.2补肾填精法治疗老年性痴呆的物质基础研究 3](#_Toc409529959)

[2.病例入选 3](#_Toc409529960)

[2.1诊断标准 3](#_Toc409529961)

[2.1.1老年性痴呆（AD）诊断标准 3](#_Toc409529962)

[2.1.2中医证候诊断标准（中华人民共和国标准化中医临床诊疗术语-证候部分GB/T 16751.2-1997） 4](#_Toc409529963)

[2.2纳入标准 4](#_Toc409529964)

[2.3排除标准 4](#_Toc409529965)

[2.4受试者退出试验的条件和步骤 5](#_Toc409529966)

[2.4.1研究者决定的退出 5](#_Toc409529967)

[2.4.2受试者自行退出试验 5](#_Toc409529968)

[2.5剔除病例标准 5](#_Toc409529969)

[3.研究方法 5](#_Toc409529970)

[3.1整体设计 5](#_Toc409529971)

[3.2病例分配 5](#_Toc409529972)

[3.3治疗方案 6](#_Toc409529973)

[3.4疗程 6](#_Toc409529974)

[3.5随访时点 6](#_Toc409529975)

[3.6研究用药管理制度 6](#_Toc409529976)

[3.6.1研究前管理 6](#_Toc409529977)

[3.6.2研究期管理 6](#_Toc409529978)

[3.6.3供药方式 7](#_Toc409529979)

[3.6.4研究后管理 7](#_Toc409529980)

[4.辅助检查 7](#_Toc409529981)

[4.1动态检测指标 7](#_Toc409529982)

[4.1.1检测指标： 7](#_Toc409529983)

[4.1.2采血时点： 7](#_Toc409529984)

[4.1.3检测指标相关材料： 7](#_Toc409529985)

[4.2 BOLD-fMRI、波谱成像、海马体积测定 8](#_Toc409529986)

[4.2.1检查单位： 8](#_Toc409529987)

[4.2.2留存资料： 8](#_Toc409529988)

[4.2.3检查操作： 8](#_Toc409529989)

[5. 不良事件与处理 10](#_Toc409529990)

[5.1记录与报告 10](#_Toc409529991)

[5.2病人的处理 10](#_Toc409529992)

[6. 临床研究质量控制与保证 11](#_Toc409529993)

[6.1研究者注意事项 11](#_Toc409529994)

[6.2保证受试者的依从性 11](#_Toc409529995)

[6.3实验室质量控制 11](#_Toc409529996)

[6.4建立多中心协调委员会 11](#_Toc409529997)

[6.5制定临床监查员制度 12](#_Toc409529998)

[7.数据管理 12](#_Toc409529999)

[7.1病例报告表的填写与移交 12](#_Toc409530000)

[7.2数据的录入与修改 12](#_Toc409530001)

[7.3数据锁定与处理 12](#_Toc409530002)

[8. 课题保密 13](#_Toc409530003)

# 1.研究内容

本研究设补肾中药（选方均为中医经验方-淫羊藿、补骨脂、制首乌、黄芪、川芎、女贞子、石菖蒲(深圳市三九现代中药有限公司提供)等，经过长期临床实践验证，疗效肯定，安全可靠）及口服治疗老年痴呆的盐酸多奈哌齐(商品名：安理申，卫才（中国）药业有限公司提供，5mg/片)，开展多中心、随机、平行对照临床研究，以验证补肾方对肾虚髓亏证AD的有效性。

## 1.1补肾填精法治疗老年性痴呆临床疗效评价研究

通过中医证候积分、老年性痴呆相关评价量表、影像学特异性表现等检测手段，评价滋补肾精法治疗老年性痴呆的临床疗效；通过长期随访量表评价及终点事件调查，评价该法治疗老年性痴呆的远期疗效，以阐释滋补肾精法治疗老年性痴呆肾虚髓亏证的证治规律，揭示“肾生髓，脑为髓海”与老年性痴呆临床疗效的内在联系，深化从肾论治老年性痴呆的临床指导意义。

## 1.2补肾填精法治疗老年性痴呆的物质基础研究

1.2.1血浆特异性蛋白表达等相关指标检测

1.2.2老年性痴呆患者“NEI网络”相关指标检测

# 2.病例入选

## 2.1诊断标准

### 2.1.1老年性痴呆（AD）诊断标准

按照美国精神病协会《美国精神病学会的精神障碍诊断手册和统计工作手册标准，第四版，1994年修订》（DSM-IV）中有关痴呆的诊断标准：

A．发生多方面认知缺陷，表现为下列二者：

（l）记忆缺损（学习新信息的能力缺损或不能回忆以前所学到的信息）；

（2）至少下列认知障碍之一：

（a）失语（语言障碍）；

(b）失用（虽然运动功能没有问题，但不能执行动作）；

（C）失认（虽然感觉功能没有问题，但不能认识或识别物体）；

（d）执行管理功能的障碍（即：计划、组织、按排次序、抽象）。

B．符合 A1与 A2的认知缺陷导致社交或职业功能的缺损，并可发现这些功能明显不如以前。

C．病程的特点是逐渐起病，继续减退。

D．符合A1与A2的认知缺陷，并非由于下列原因：

（l）其他能导致记忆与认知进行性缺陷的中枢神经系情况（例如，心血管疾病、巴金森病、享丁顿病、硬膜下血肿、正常压力膨积水、脑瘤）；

（2）已知能导致痴呆的系统性情况（例如，甲状腺功能减退、维生素B12或叶酸缺

乏、烟酸缺乏、低血钙、神经梅毒、HIV感染）；

（3）物质所致情况。

E．这些缺陷并非由于谵妄所致。

F．此障碍并非由于其他轴I障碍所致（例如重性抑郁、精神分裂症）。

### 2.1.2中医证候诊断标准（中华人民共和国标准化中医临床诊疗术语-证候部分GB/T 16751.2-1997）

肾虚髓亏证：肾精亏虚，精髓不足，以生长发育迟缓，或骨折久不愈合，或腰酸骨痿，头晕耳鸣，健忘痴呆等为常见症的证候。

## 2.2纳入标准

（1）符合痴呆及老年性痴呆诊断标准（采用DSM-IV标准）；

（2）中医证候诊断为肾虚髓亏证；

（3）年龄60-85岁；

（4）HIS≤4分；

（5）HAMD≤7分；

（6）受试者和/或其监护人签署知情同意书；

（7）CDR=1.0。

## 2.3排除标准

（1）患有血管性痴呆或其他原因所致的痴呆者；

（2）合并严重的心、肝、肾和造血系统疾病（窦性心动过缓及传导阻滞、AST、ALT超过正常值上限2倍者；肾功能检查BUN超过正常值上限1.5倍，Cr超过正常值范围者）；

（3）过敏体质或对盐酸多奈哌齐、哌啶衍生物过敏者；

（4）近一个月内使用任何可能影响认知功能的药物史；

（5）经药物治疗未能控制的高血压（收缩压大于180mmHg或舒张压高于100mmHg）；

（6）具有干扰认知测验的疾患如失语、偏瘫及其他（如严重的听觉、视觉缺陷，言语交流困难等）；

（7）有晕厥史、哮喘史及慢性阻塞性肺病史者；

（8）正在参加其他药物临床试验者；

（9）病情危重，难以对研究用药的有效性和安全性做出确切评价者。

## 2.4受试者退出试验的条件和步骤

### 2.4.1研究者决定的退出

受试者退出试验是指已经入选的受试者在试验过程中出现了不宜继续进行试验的情况下，研究真决定该病例退出试验。

### 2.4.2受试者自行退出试验

根据知情同意书的规定，受试者有权中途退出试验，或受试者虽未明确提出退出试验，但不再接受用药及检测而失访，也属于“退出”（或称“脱落”）。应尽可能了解其退出的原因，并加以记录。无论何种原因，对退出试验的病例应保留其病例报告表，并以其最后一次随访或检测结果转为最终结果，对其疗效和不良反应进行全数据集分析。

## 2.5剔除病例标准

（1）严重违反纳入标准的病例。

（2）纳入后未曾用药或无任何可评价记录的病例。

# 3.研究方法

## 3.1整体设计

采用分层随机、平行对照、多中心临床试验设计方法，由天津中医药大学第二附属医院脑病临床研究中心负责随机化方案的设计。

## 3.2病例分配

采用多中心临床研究方法，研究中心共三家，天津中医药大学第二附属医院观察40例，天津环湖医院观察20例，上海中医药大学附属龙华医院观察60例，各中心纳入试验组与对照组按1:1计算，共观察120例符合纳入标准的病例。各临床研究中心应严格按照本方案规定的各项标准选择病例。

## 3.3治疗方案

中药组予淫羊藿（10g）、补骨脂（10g）、制首乌（10g）、炙黄芪（10g）、川芎（6g）、女贞子（10g）、石菖蒲（6g）免煎颗粒剂（深圳市三九现代中药有限公司提供）各1袋，每次半袋，饭后半小时温开水冲服，每日2次。西药组予盐酸多奈哌齐（商品名：安理申，卫才（中国）药业有限公司提供，5mg/片）5mg/次，睡前服，1次/日。

注：入组病例均不能服用任何其他益智类中西药及采取任何影响认知功能评价的治疗措施。

## 3.4疗程

自入组之日起，服药24周。

## 3.5随访时点

（1）认知功能相关量表：入组（0天）、治疗后12周、治疗后24周、治疗后48周。

（2）影像学检查（BOLD-fMRI、波谱成像、海马体积测定）：入组（0天）、治疗后24周。

（3）NEI网络指标：入组（0天）、治疗后4周、治疗后24周。

（4）安全性指标：入组（0天）、治疗后12周、治疗后24周。

（5）AD相关特异性理化检查：入组（0天）、治疗后24周。

## 3.6研究用药管理制度

### 3.6.1研究前管理

研究前由课题负责单位准备试验所需用药，并分发给各临床研究中心。

### 3.6.2研究期管理

建立研究药品管理制度，观察医师在受试者签署知情同意书后，按入组顺序确定随机号，拆开随机信封，确定组别后分别给予不同试验药品。药品专柜上锁，室温保存。每次发药时药品管理员于药品发放登记本上记录发药日期、受试者姓名、随机号、组别、给药数量、剩余药品数量，并签名。

### 3.6.3供药方式

初次供药将根据以往研究工作估算用量，邮寄给各临床研究中心，如课题进行中各研究中心剩余1/4药量时，请根据研究进度，及时联系课题负责单位，以决定是否需要补发药品。

### 3.6.4研究后管理

每个临床研究中心将剩余药品集中返还课题负责单位。

# 4.辅助检查

## 4.1动态检测指标

### 4.1.1检测指标：

（1）安全性指标：血、尿、便常规，心电图，ALT、AST、BUN、Cr

（2）疗效性指标：NEI网络指标（促肾上腺皮质激素、血浆皮质醇、生长激素、雌二醇、睾酮、儿茶酚胺、乙酰胆碱、血管活性肠肽、促甲状腺素；CD3+、CD4+、CD8+细胞比例，CD4+/CD8+之比；IL-2、IFNγ、TGF、IL-1），T-tau、P-tau、Aβ1-42

### 4.1.2采血时点：

（1）安全性指标：入组（0天）、治疗后12周、治疗24周后

（2）疗效性指标：NEI网络指标：入组（0天）、治疗4周后、治疗24周后

T-tau、P-tau、Aβ1-42：入组（0天）、治疗24周后

### 4.1.3检测指标相关材料：

（1）安全性指标各中心按要求分别完成，常规取血检测。

（2）疗效性指标由上海中医药大学附属龙华医院承担。

## 4.2 BOLD-fMRI、波谱成像、海马体积测定

### 4.2.1检查单位：

由天津中医药大学第二附属医院承担

### 4.2.2留存资料：

将纳入病例检查所得影像资料以照片、纸质及刻录成光盘方式保存。

### 4.2.3检查操作：

（1）图像采集

机器：Siemens Trio 3.0T 超导型全身磁共振成像系统，采用标准正交头线圈，采集序列如下：

常规T1WI、T2WI、FLAIR 图像：用于排除器质性病变、脑血管疾病等，采用临床扫描常规序列，不对扫描参数进行界定。

解剖像：3D 梯度回波T1 加权序列，矢状位图像，176 层，TR/TE=1900/2.5ms，反转角9°，层厚1.0mm，FOV=250mm×250mm，矩阵=256×256。

脑功能图像：EPI 序列，TR/TE= 3000/30ms，反转角为90°，层厚4.0mm，间隔1.0mm，共25 层，FOV=220mm×220mm，矩阵=64×64，扫描时间为12min。扫描方位平行于前后联合，共采集240 个时间点。

海马体积测量：采用解剖像三维数据进行海马轮廓勾勒、分割、计算扣带回下部、颞叶皮层、颞叶单体素MRS 测量：TR：2000ms，TE：30ms，2048 数据点，激发次数：128，感兴趣容积：8cm3(2×2×2cm)。

（2）脑功能成像试验刺激方案

采用E-PRIME 软件设计刺激任务，对病人进行扫描前训练，扫描过程中采用同步触发脑功能成像任务呈现系统呈现刺激，并记录病人反映情况。病人通过正交线圈上方固定的标准反射镜观察图像呈现屏呈现的刺激图像，刺激源距受试者双眼约0.6米。扫描期间进行头部固定，减小非自主头动的影响。扫描层面与前颅底平行，即前联合－后联合连线，眼球与幕上结构不在一个层面上，避免不自主眼动对脑激活信号的影响。

实验设计采用改良的组块刺激方案，即基线→任务(一个组块)反复进行的模式，共6个组块，每个组块包括基线20帧图像和任务20帧图像，每帧图像采集时间为3秒，一个组块2分钟，共计12分钟。刺激方案内容为汉语stroop任务，三个组块为字色一致(即红字印红色，蓝字印蓝色，绿字印绿色)任务，三个组块为字色冲突(即绿字印成红色，红字印成蓝色，蓝字印成绿色等)任务，其中字色一致任务组块伪随机的方式放入3三个字色冲突任务，字色冲突任务组块以伪随机的方式放入3个字色一致任务。数据分析可以采用组块设计方案进行分析，亦可以采用事件相关设计方案进行分析，本研究伪随机放入冲突任务较少，采用组块分析方案。

（3）数据后处理

所有数据均采用基于matlab7.1的脑功能成像统计参数图软件5.0(spm5.0)进行离线处理和统计分析。步骤如下：将机器扫描图像导出为dicom图像采用mricron软件进行格式转换，转换为nifti格式时间点校正，根据扫描采集方式及感兴趣层面进行时间点校正头动校正，根据采集图像帧之间的位置变化信息，估计头动参数，根据头动参数进行重采样图像配准，把低分辨的功能像数据和高分辨的解剖像数据采用互信息的方法实现图像配准，按照功能像的空间信息对解剖像数据进行重新采样空间标准化：根据图像配准后的解剖像数据估计空间标准化过程中的形变信息，根据此形变信息对图像配准后的解剖像和功能像进行空间标准化图像平滑：采用5mm高斯平滑核对空间标准化后的功能像数据进行卷积运算，减小图像噪声，提高数据正态性根据刺激呈现方案，在一次统计过程中输入试验设计方案，输入相应功能数据，获得激活图图像采用单因素方差分析方法，比较两组受试者脑功能激活差异，采用配对t检验的方法，观察受试者服药前后脑功能改变情况，采用独立样本t 检验方法，比较AD病人服药前后脑激活改变的差异情况，并与病人临床资料、认知测试评分、单体素MRS测量代谢物信息进行相关分析导出激活体素的座标，体素数目和激活强度信息三维图谱叠加显示激活体素及配对t检验所示组间差异体素的空间定位信息MRS获得代谢物(包括NAA (2.0 ppm)、Cr(3.0 ppm)、Cho(3.2 ppm)和MI(3.56 ppm)信息，计算出MI/Cr、NAA/Cr、Cho/Cr 和MI/NAA。根据数据的正态性，进行单因素方差分析或者多组独立样本非参数检验的秩和检验。

# 5. 不良事件与处理

不良事件是指病人或临床试验的受试者接受一种药品后出现的不良医学事件，但不一定与治疗有因果关系。也包括研究开始前存在的疾病发病次数和严重程度的增加。严重不良事件是指临床试验过程中发生需住院治疗、延长住院事件、伤残、影响工作能力、危及生命或死亡、导致先天畸形等事件。

## 5.1记录与报告

研究者向患者说明，要求患者如实反映用药后的病情变化。医生避免诱导性提问。在观察疗效同时，注意观察不良反应。无论不良反应或不良事件是否与本研究用药相关，均应详细记录，包括不良反应出现时间、症状、体征、程度及发作频度、持续终止时间、实验室检查指标、处理方法与结果、经过、随访时间等。并应详细记录合并用药的情况，以便分析不良反应与试验药物的相关性。是否需要治疗，如需要，请记录所给予的治疗。研究者判断不良事件是否与应用试验药品有关。随访选择住院、门诊、家访、电话等形式。

如果发生严重不良事件，研究者必须在24小时之内或不迟于第二个工作日向临床研究负责单位报告。研究者要在报告上签名并注明日期，在原始资料中记录何时、以何种方式、向谁报告了严重不良事件。

## 5.2病人的处理

发现不良反应时，研究者可根据病情采取必要的处理措施，如：调整试验用药剂量、暂时中断用药、永久性停药等。出现严重不良事件，承担临床研究的单位必须立即采取必要处理措施，保护受试者安全。所有不良事件都应当追踪调查，详细记录处理经过及结果，直到得到妥善解决或病情稳定，若化验异常者应追踪至恢复正常。追踪随访方式可以根据不良反应的轻重选择住院、门诊、家访、电话、通讯等多种形式。

# 6. 临床研究质量控制与保证

## 6.1研究者注意事项

（1）参加临床研究的研究者要相对固定，并具有临床研究的专业特长、资格和能力。

（2）临床研究开始前，组织参加临床研究的医师认真学习临床研究方案，落实各项技术指标。

（3）受试者按观察方案的要求复诊，如病人未能按时复诊，研究医师及时通知其复诊或进行登门随访。

## 6.2保证受试者的依从性

临床研究中，为保证受试者的依从性，应使受试者充分理解本研究的意义及按时服药的重要性。

（1）要求受试者按时服药，并由家属督促。

（2）采用药物计数法，随访时，要求受试者必须将剩余用药返还，由研究者清点药品数量，对未完全按照要求服药的原因要详细记录，对疗效差或依从性差的受试者要加强监督。

（3）受试者依从性评价：依从性=（实际服药量/应该服药量）*100%

依从性良好：依从性80%-120%

## 6.3实验室质量控制

（1）安全性指标检测分别见各中心临床检验操作规范（三个中心均为三级甲等医院）；

（2）NEI网络及特异性蛋白检测操作规范详见附册（由上海中医药大学附属龙华医院负责）

## 6.4建立多中心协调委员会

由牵头单位总负责，组织各临床研究中心组成临床协调委员会，委员会的主要职责是协调整个临床研究的实施，解决临床研究中出现的有关问题。

## 6.5制定临床监查员制度

由课题负责单位派监查员，监督临床研究单位实施的全过程。

# 7.数据管理

## 7.1病例报告表的填写与移交

每位受试者纳入后3天内，观察医师将受试者《病例报告表》第7-8页（即受试者一般资料、病史等）复印后传真至课题负责单位-天津中医药大学第二附属医院，以保证信息及时汇总分析。传真号：022-60335229。

研究者根据受试者的原始观察记录，将数据及时、完整、正确、清晰地载入病例报告表。出组3天内必须完成病例报告表。完成的病例报告表由临床监查员审查后，移交数据管理员，进行数据录入与管理工作。

## 7.2数据的录入与修改

数据录入与管理由数据管理员负责。数据管理员应与主要研究者一起，按病例报告表中各指标数值的范围和相互关系拟定数据范围检查和逻辑检查内容，并编写相应的计算机程序，在输入前控制错误数据输入，所有错误内容及修改结果应有记录并妥善保存。为保证数据的真确性，应由两个数据管理员独立进行双份录入并校对。对病例报告表中存在的疑问，数据管理员将产生疑问解答表（DRQ），并通过临床监查员向研究者发出询问，研究者应尽快解答并返回，数据管理员根据研究者的回答进行数据修改、确认与录入，必要时可以再次发出疑问解答表。

## 7.3数据锁定与处理

在审核并确认建立的数据库正确后，由课题负责单位人员、统计分析人员对数据进行锁定。锁定后的数据文献不再做改动。数据锁定之后发现的问题，经确认后在统计分析程序中进行修正。将数据库交统计分析人员按统计计划书要求进行统计分析。完成统计分析后，由统计分析人员撰写统计分析报告，交课题负责单位研究人员写出研究报告。

# 8. 课题保密

各临床研究中心人员要注意课题资料的保密，不得将病例调查资料转交他人使用。
