# Supplementary material for: Cognitive Improvement during Treatment for Mild Alzheimer’s Disease with a Chinese Herbal Formula: A Randomized Controlled Trial
Source: PLoS One. 2015 Jun 15;10(6):e0130353. doi: 10.1371/journal.pone.0130353 (PMC4468068; doi:10.1371/journal.pone.0130353)
Supplement: S2 Trial Protocol — (DOCX) [file pone.0130353.s009.docx]

**Trial Protocol**

**The Theory Study of “The Kidney Stores Essence and Brain Is the Sea of Marrow” for AD**

Major Project of Chinese National Programs for Fundamental Research and Development (973 Program) ( 2010CB530405).

**ChiCTR-TRC-12002846**

***A randomised, double-blind, double dummy, parallel controlled trial of Chinese herbs in the treatment of the Alzheimer’s disease***

**Funding:** Ministry of Science and Technology of China

**Target disease:** Alzheimer’s disease

**Study type:** Interventional

**Sponsor:**  Second Affiliated Hospital of Tianjin University of TCM

**Study leader**: Yulian Zhang

**Protocol Date**: 1st Jan 2010

**Study execute time:** From2010-1-1 to 2014-12-31

**Recruitment and research settings**: The second hospital affiliated toTianjin university of traditional Chinese medicine

Longhua Hospital SHanghai University of TCM

Tianjin Huanhu Hospital

**Principal Investigator**: **Dr Yulian Zhang**

816 Zhenli Road, Hebei District Tianjin,

Tel. +86-13821151196

Email: zhyl220@126.com

The trial will be conducted in compliance with the protocol and other regulatory requirements as appropriate.

contents

[Abbreviation 5](#_Toc409528768)

[1 Introduction 6](#_Toc409528769)

[**1.1** **Clinical efficacy evaluation of tonifying kidney essence method in treating AD** 6](#_Toc409528770)

[**1.2** **Material basis of tonifying kidney essence method in treating AD** 7](#_Toc409528771)

[2 Patient 7](#_Toc409528772)

[**2.1 Diagnostic criteria** 7](#_Toc409528773)

[2.1.1 Alzheimer’s disease 7](#_Toc409528774)

[2.1.2 Clinic terminology of traditional Chinese medical diagnosis treatment-Syndrome(GB/T 16751.2-1997) 9](#_Toc409528775)

[**2.2 Inclusion criteria** 9](#_Toc409528776)

[**2.3Exclusion criteria** 10](#_Toc409528777)

[**2.4 Withdrawing rules** 11](#_Toc409528778)

[2.4.1 Withdrawn by the researchers 11](#_Toc409528779)

[2.4.2 The patient decides to withdraw from the study 11](#_Toc409528780)

[**2.5 exclusion cases standard** 11](#_Toc409528781)

[3 Methods 11](#_Toc409528782)

[**3.1 Study design** 11](#_Toc409528783)

[**3.2 Patient enrollment** 12](#_Toc409528784)

[**3.3 Intervention** 12](#_Toc409528785)

[**3.4 Treatment duration** 13](#_Toc409528786)

[**3.5 Time point** 13](#_Toc409528787)

[**3.6 Drug monitoring** 13](#_Toc409528788)

[3.6.1 Study preparing 13](#_Toc409528789)

[3.6.2 Studying 14](#_Toc409528790)

[3.6.3 Drugs supplying 14](#_Toc409528791)

[3.6.4 Study finished 14](#_Toc409528792)

[4 Auxiliary examination 14](#_Toc409528793)

[**4.1 Dynamic outcomes** 15](#_Toc409528794)

[4.1.1 Detection outcomes 15](#_Toc409528795)

[4.1.2 Sampling point 15](#_Toc409528796)

[4.1.3 Related materials 15](#_Toc409528797)

[**4.2 BOLD-fMRI, magnetic resonance spectroscopy, hippocampus formation** 16](#_Toc409528798)

[4.2.1 Performed 16](#_Toc409528799)

[4.2.2 Data saving 16](#_Toc409528800)

[4.2.3 Operation 16](#_Toc409528801)

[5 Adverse event 20](#_Toc409528802)

[**5.1 recording and reporting** 21](#_Toc409528803)

[**5.2 Follow up of AEs** 22](#_Toc409528804)

[6 Quality assurance 22](#_Toc409528805)

[**6.1 Investigators** 22](#_Toc409528806)

[**6.2 Compliance** 23](#_Toc409528807)

[**6.3 Laboratory test** 24](#_Toc409528808)

[**6.4 Muti-center coordinating committee** 24](#_Toc409528809)

[**6.5 Monitors** 24](#_Toc409528810)

[7 Data management 24](#_Toc409528811)

[**7.1 Case report form (CRF)** 24](#_Toc409528812)

[**7.2 Data entry and modification** 25](#_Toc409528813)

[**7.3 Data processing** 25](#_Toc409528814)

[8 Confidentiality 26](#_Toc409528815)

# Abbreviation

AD Alzheimers disease

ADAS-cog Alzheimer’s Disease Assessment Scale-cognitive subscale

AE Adverse Event

CDR Clinical Dementia Rating

COPD Chronic obstructive pulmonary disease

CRF Case report form

DSM-IV Dementia of the Alzheimer's type in Diagnostic and Statistical Manual of Mental Disorders, 4th edition

HAMD Hamilton Anxiety Scale

HIS Hachinski Ischemic Score

MMSE Mini-mental state examination

MRI Magnetic resonance image

NPI Neuropsychiatric Inventory

SAE Serious Adverse Event

TCM Traditional Chinese medicine

# Introduction

We conducted a 48 weeks, randomized, double-blind, double-dummy, and multicenter trial to explore the efficacy in improving cognitive function in patients with mild Alzheimer’s disease(AD) of kidney and marrow deficiency syndrome by Chinese herbal formula to tonify the kidney, company with donepezil hydrochloride (DH) (provided by Eisai China Inc. Tianjin, China). The formula is composed of Yinyanghuo (Epimedium), Nvzhenzi(Fructus Ligustri Lucidi),Buguzhi (Psoralea fruit), Heshouwu (Radix Polygoni Multiflori), Huangqi (Radix Astragali), Chuanxiong(Ligusticum wallichi Franchat), Shichangpu(Acorus gramineus)(provided by Shenzhen Sanjiu Modern Chinese Medicine limited Company, Tianjin, China).

## **Clinical efficacy evaluation of tonifying kidney essence method in treating AD**

To evaluate the clinical efficacy of tonifying kidney essence method in treating AD by total scores of traditional Chinese medical syndrome, Alzheimer’s Disease Assessment Scale-cognitive subscale (ADAS-cog), Mini-Mental State Examination (MMSE), Activity of Daily Living Scale (ADL), Neuropsychiatric Inventory (NPI), and magnetic resonance image(MRI); to evaluate the long term efficiency of tonifying kidney essence method in treating AD through investing the follow-up and end point. We try to reveal the rules of tonifying kidney essence method in treating AD and the relationship between the theory “The Kidney Stores Essence and Brain Is The Sea of Marrow” and AD, in order to set guidelines for treating AD from kidney.

## **Material basis of tonifying kidney essence method in treating AD**

1.2.1specific plasma proteins expression related outcome test

- - 1. “NEI network” related outcome test

# Patient

## **2.1 Diagnostic criteria**

### 2.1.1 Alzheimer’s disease

According to the Diagnostic criteria for 294.1x Dementia of the Alzheimer's type in Diagnostic and Statistical Manual of Mental Disorders, 4th edition (DSM-IV) (APA, 1994)：

A. The development of multiple cognitive deficits manifested by both

(1) memory impairment (impaired ability to learn new information or to recall previously learned information)

(2) one (or more) of the following cognitive disturbances

(a) aphasia (language disturbance)

(b) apraxia (impaired ability to carry out motor activities despite intact motor function)

(c) agnosia (failure to recognize or identify objects despite intact sensory function)

(d) disturbance in executive functioning (i.e., planning, organizing, sequencing, abstracting )

B. The cognitive deficits in Criteria A1 and A2 each cause significant impairment in social or occupational functioning and represent a significant decline froma previous level of functioning.

C. The course is characterized by gradual onset and continuing cognitive decline.

D. The cognitive deficit in Criteria A1 and A2 are not due to any of the following:

(1) other central nervous system conditions that cause progressive deficits in memory and cognition( e.g., cerebrovascular disease, Parkinsin's disease, Huntington's disease, subdural hematoma, normal-pressure hydrocephalus, brain tumor)

(2) systemic conditions that are known to cause dementia (e.g., hypothyroidism, vitamin B_12_ or folic acid deficieney, niacin deficiency, hypercalcemia, neurosyphilis, HIV infection)

(3) substance-induced conditions

E.The deficits do not occur exclusively during the course of delirium.

F. The disturbance is not better accounted for by another Axis I disorder(e.g., Major Depressive Disorder, Schizophrenia).

### 2.1.2 Clinic terminology of traditional Chinese medical diagnosis treatment-Syndrome(GB/T 16751.2-1997)

Syndrome of kidney and marrow deficiency: kidney-essence and marrow deficiency manifests as growth retardation, long nonunion fracture, aching lumbar, dizziness, tinnitus, obliviousness and dementia.

## **2.2 Inclusion criteria**

(1) a diagnosis of dementia of the Alzheimer’s type according to the Diagnostic and Statistical Manual of Mental Disorders, 4th edition (DSM-IV) (APA, 1994);

2) a diagnosis of Syndrome of kidney and marrow deficiency

(3) women or men aged 50–85 years;

(4) Hachinski Ischemic Score(HIS) ≤4;

(5) Hamilton Anxiety Scale(HAMD) score of ≤7;

(6)Written informed consent from all patients (or their legal representatives);

(7) Clinical Dementia Rating (CDR) score of 1.

## **2.3Exclusion criteria**

(1) with vascular dementia or any neurological disorder other than AD that contributed substantially to dementia;

(2) with severe heart, liver, kidney and blood system diseases (sinus bradycardia and atrioventricular block, AST and ALT 2 times more than the upper limit of normal value; kidney function tests showing BUN 1.5 times higher than the upper limit of normal value, Cr more than the normal value );

(3)allergies or allergic to donepezil hydrochloride and Piperidine Derivatives;

(4) the use of any drugs that may affect cognitive function 4 weeks prior to randomization;

(5) uncontrolled hypertension;

(6) with diseases that may interfere with cognitive tests such as aphasia, hemiplegia, and others(severe visual or hearing loss);

(7) with serious complications (asthma and chronic obstructive pulmonary disease(COPD));

(8) participation in another investigational new drug trial;

(9) with advanced, severe disease that could interfere with study assessments.

## **2.4 Withdrawing rules**

### 2.4.1 Withdrawn by the researchers

If further participation is a health risk for the patient, the researcher decides the patient should terminate.

### 2.4.2 The patient decides to withdraw from the study

根据知情同意书的规定，受试者有权中途退出试验，或受试者虽未明确提出退出试验，但不再接受用药及检测而失访，也属于“退出”（或称“脱落”）。应尽可能了解其退出的原因，并加以记录。无论何种原因，对退出试验的病例应保留其病例报告表，并以其最后一次随访或检测结果转为最终结果，对其疗效和不良反应进行全数据集分析。

According to the informed consent provisions, the patient has the right to withdraw from the trial; If patients withdraw their consent for follow-up then, if at all possible, the reason for loss to follow-up should be ascertained and permission should be requested to use data collected already for the purposes of the trial.

## **2.5 exclusion cases standard**

（1）严重违反纳入标准的病例。

(1) a serious breach of the inclusion criteria.

（2）纳入后未曾用药或无任何可评价记录的病例。

(2) no medication intake or no case evaluation recorded after screened.

# 3 Methods

## **3.1 Study design**

The randomized, double-blind, double-dummy, parallel controlled and multicenter trial designed by Cerebropathy Study Center in the Second Affiliated Hospital of Tianjin University of TCM.

## **3.2 Patient enrollment**

Patients are recruited from three centers, 40 in the Second Affiliated Hospital of Tianjin University of TCM, 20 in the Tianjin Huanhu Hospital, and 60 in the Longhua Hospital of Shanghai University of TCM, in a 1:1 ratio.

## **3.3 Intervention**

The TCM formula in our study was YHD, including Yinyanghuo (Epimedium) 10g, Nvzhenzi(Fructus ligustri lucidi) 10g, Buguzhi (Psoralea fruit) 10g, Heshouwu (Radix polygoni multiflori)10g, Huangqi (Radix astragali) 10g, Chuanxiong(Ligusticum wallichi franchat) 6g, Shichangpu(Acorus gramineus) 6g in one unit. Each herb was provided as herbal concentrate-granules in one bag and quality controlled by the Shenzhen Sanjiu Modern Chinese Medicine limited Company (Tianjin, China). Every patient in YHD group orally took 100 ml of the decoction once a day half hour after breakfast and DH-simulation 5 mg before sleep for 24 weeks. Patients in DH group orally took DH 5 mg a day before sleep provided by Eisai China Inc. (Tianjin, China), and YHD-simulation 100 ml of the decoction half hour after breakfast for 24 weeks.

## **3.4 Treatment duration**

It’s a 24 weeks treatment trial.

## **3.5 Time point**

(1) Cognitive scales: baseline (0 week), week 12(treatment), week 24(treatment), week -48(follow up);

(2) Imaging (BOLD-fMRI, magnetic resonance spectroscopy, hippocampus formation): baseline (0 week), week 12(treatment);

(3) NEI network: baseline (0 week), week 4(treatment), week 24(treatment);

(4) Safety outcomes: baseline (0 week), week 12(treatment), week 24(treatment)**;**

(5) Specific parameters related to AD: baseline (0 week), week 12(treatment).

## **3.6 Drug monitoring**

### 3.6.1 Study preparing

All the drugs are provided by the amenable administrators for the study centers.

### 3.6.2 Studying

After the participants sign informed consent, the researcher set the random number according to the sequence of recruitment; open the randomized envelope and give different drugs to patients in different groups. The drugs are locked in special Counter and stored at room temperature. The drug administrator should record the data, name of patient, random number, group, dosage, and residual drugs every distribution.

### 3.6.3 Drugs supplying

The first drug supply will be estimated for consumption based on the previous research work, and mailed to all clinical study centers. If the drugs are used with residual 1/4 in the study center, they should timely contact with the unit in charge, to determine the need for replacement drugs according to the study schedule.

### 3.6.4 Study finished

Each clinical research center will return the remaining drug to the sponsor.

# Auxiliary examination

## **4.1 Dynamic outcomes**

### 4.1.1 Detection outcomes

（1）安全性指标：血、尿、便常规，心电图，ALT、AST、BUN、Cr

(1) safety outcomes: blood, urine, stool routine, electrocardiogram, ALT, AST, BUN, Cr（2）疗效性指标：NEI网络指标（促肾上腺皮质激素、血浆皮质醇、生长激素、雌二醇、睾酮、儿茶酚胺、乙酰胆碱、血管活性肠肽、促甲状腺素；CD3+、CD4+、CD8+细胞比例，CD4+/CD8+之比；IL-2、IFNγ、TGF、IL-1），T-tau、P-tau、Aβ1-42

(2) outcomes of curative effect: NEI network index (adrenocorticotropic hormone, cortisol, growth hormone, estradiol, testosterone, catecholamine, acetylcholine, vasoactive intestinal peptide, thyroid stimulating hormone; CD3+, CD4+, CD8+ , CD4+/CD8+ ; IL-2, IFNγ, TGF, IL-1), T-tau, P-tau,Aβ1-42

### 4.1.2 Sampling point（1）安全性指标：入组（0天）、治疗后12周、治疗24周后

(1) safety indicators: baseline (0 week), week 12(treatment), week 24(treatment);（2）疗效性指标：NEI网络指标：入组（0天）、治疗4周后、治疗24周后

(2) outcomes of curative effect:

NEI: baseline (0 week), week 4(treatment), week

24(treatment);T-tau、P-tau、Aβ1-42：入组（0天）、治疗24周后

T-tau, P-tau, Aβ1-42: baseline (0 week), week 24(treatment);

### Related materials

（1）安全性指标各中心按要求分别完成，常规取血检测。

(1) Safety outcomes should be performed in each center.

（2）疗效性指标由上海中医药大学附属龙华医院承担。

(2) Index of curative effect should be performed by Longhua hospital affiliated to Shanghai University of TCM.

## **4.2 BOLD-fMRI, magnetic resonance spectroscopy, hippocampus formation**

### 4.2.1 Performed

In the Second Affiliated Hospital of Tianjin University of TCM.

### 4.2.2 Data saving

The images are saved as photos, papers, and discs.

### 4.2.3 Operation

**(1) Image acquiring**

MRI data were acquired on a Siemens Trio scanner (SiemensMedical Systems, Erlangen, Germany) at 3 T, using a standard orthogonal coil.

Acquisition sequence: T1WI, T2WI, FLAIR image: used to exclude organic disease, cerebrovascular disease, using clinical scanning routine sequence, without defined scanning parameters.

Anatomical study: in a 3D gradient recalled echo T1 weighted image, sagittal images, 176 layer, TR/TE=1900/2.5ms, 9 degrees flip angle, slice thickness 1.0mm,FOV=250mm * 250mm, matrix =256 * 256.

Brain functional images: EPI series, TR/TE= 3000/30ms, 90 degree flip angle, slice  thickness 4.0mm, 1.0mm interval, a total of 25 layers, FOV=220mm * 220mm, matrix =64 * 64 , with the scanning time of 12min. The scanning range is parallel to the posterior, collecting a total of 240 time points.

Hippocampal volume measurement: the anatomical image 3-D data is performed to outline and segment the hippocampus, calculate cingulate cortex, cortex of temporal lobe, and temporal lobe single voxel. MRS measurements: TR: 2000ms, TE: 30ms, 2048 data points; stimulation: 128 times, the volume of interest: 8cm3 (2 * 2 * 2cm).

**(2) Functional brain imaging stimulus**

The stimulus is designed by E-PRIME. The patient will be trained before scanning, and the synchronous trigger brain functional imaging task presentation system is performed to present the stimuli. The patients watch the image presented on the screen through the standard reflector fixed above the orthogonal coil, and the image is 0.6 meter away from the patients. During the scanning, the head should be fixed to reduce the influence of non-independent movement. Scanning plane is parallel to anterior skull base, namely the anterior commissure and the posterior commissure are in a line. The eyes and supratentorial structure is not on the same level to avoiding the influence of involuntary eye movement on the activation signal of the brain.

We use reformed adopted block stimulus plan, a repeated baseline to task (a block) pattern. There are 6 blocks, including a baseline of 20 frame images and task 20 frame images each, three seconds per frame of the image acquisition time, two minutes a group 12 minutes in total. The stimulus for the content is Chinese Stroop task, which includes three blocks for the congruent color words ( the word red printed in red,  word blue printed in blue,  word green printed green) task, three blocks for word and color in conflict (word green printed in red, printed red in blue, word blue printed in green). Three blocks of words and color in conflict is pseudo-randomized into blocks for the congruent color words, and vice versa. The data can be analyzed using a block design, also using event related design. Regarding the less pseudo random into conflict task, we use block designe.

**(3) Data analysis**

Data analysis is carried out using brain imaging Statistical Parametric Mapping 5.0(SPM 5.0) based on matlab 7.1. The scanning image is derived for the dicom image and converted to nifti format time correction by mricron; adjust the time point according to the scan acquisition mode; estimate the head movement parameters according to the position change information between the image acquisition frame; re-sample image registration according to the head movement parameters. Imaging registration is carried by mutual information of the low resolution functional  data and high-resolution anatomical image data; resample spatial standardization of the anatomic imaging data is carried in accordance with the function of spatial information: to estimate the deformed information during the spatial standardization according to the anatomy of the data after image registration; to carry the spatial standardization imaging smoothing of  anatomical images and function image after registration according to the information of deformation. 5mm Gauss smoothing necleus is used to convolution operate the functions imaging data after standardization, reduce the image noise, and improve data normality. According to stimulus presentation scheme, input the test design scheme in a statistical process, and the corresponding function data, to get the activation map image. Single factor variance analysis method is used to compare the differences of the brain function activation between two groups; paired t test, is used to test the situation changes of brain function before and after medication; independent sample t test is used to test the difference of brain activation changes in AD patients before and after medication, and comparing to the clinical data, cognitive test scores, single voxel MRS measurement of metabolic information to derive 3D map of the activated voxel coordinate, voxel number and intensity of activation information. Metabolite MRS (including NAA (2 ppm), Cr (3 ppm), Cho(3.2 ppm) and MI (3.56 ppm))  information is obtained according to the activated voxels of the map and the between group differences in body space positioning information of paired t test  to calculate MI/Cr NAA/Cr, Cho/Cr, and MI/NAA. According to the normality of the data, single factor analysis of variance or a plurality of groups of independent samples rank sum test parameter test can be used.

# 5 Adverse event

Serious Adverse Events are defined here as:

1. **Serious Adverse Events** – those which are fatal, life threatening, disabling or require hospitalisation or prolongation of hospitalisation. All deaths whether thought to be related to the trial intervention or not should be reported as serious adverse events.

1. **Unexpected Adverse Events** – those that would not be expected among elderly patients with AD (See known side effects in BNF).

## **5.1 recording and reporting**

Care will be taken not to introduce bias when detecting AEs and/or SAEs. Open-ended and non-leading verbal questioning of the subject is the preferred method to inquire about AE occurrence. Regardless the relevance to the medicine, A it is the responsibility of the investigator to review all documentation (e.g., hospital progress notes, laboratory, and diagnostics reports) relative to the event. The investigator will then record all relevant information regarding an AE/SAE in the appropriate data collection tool.

The investigator will attempt to establish a diagnosis of the event based on signs, symptoms, and/or other clinical information. In such cases, the diagnosis will be documented as the AE/SAE and not the individual signs/symptoms.

If any patient suffers an event that fulfils these criteria, a Serious Adverse Event Form should be completed and faxed to the Principal Investigator **within 24 hours of the event being known.** The original form should be stored in the Investigator File and a copy placed in the patient’s source notes.

## **5.2 Follow up of AEs**

After the initial AE/SAE report, the investigator is required to proactively follow each subject at subsequent visits/contacts. All AEs and SAEs will be followed until resolution, until the condition stabilizes, until the event is otherwise explained, or until the subject is lost to follow-up.

The investigator is obligated to perform or arrange for the conduct of supplemental measurements and/or evaluations as may be indicated or as requested to elucidate as fully as possible the nature and/or causality of the AE or SAE. The investigator is obligated to assist. This may include additional laboratory tests or investigations, histopathological examinations or consultation with other health care professionals.

# 6 Quality assurance

## **6.1 Investigators**

(1) The researchers should be relatively permanent, and with clinical research expertise, qualification and ability.

(2) Before the trial, investigators should learn the study protocol carefully and make sure all the various technical indicators.

(3) Participants should be followed up in accordance with the protocol; if the patients fail to return on time, the physicians should inform the referral or to follow up.

## **6.2 Compliance**

In order to ensure the compliance of the participants, the participants should be made to fully understand the significance of this study and the importance of taking medicine on time.

(1) The participants should be asked to take medicine on time by caregivers.

(2) The drugs should be counted and the patients should return the remaining drug at follow up. Researchers should count the number of drugs, record the detailed reasons for incompletely intake, and strengthen the monitoring of poor efficacy or poor compliance of the patients.

(3) Compliance evaluation: compliance = (the actual dose / planed dose) *100%

good compliance: 80%-120% compliance

## **6.3 Laboratory test**

(1) Safety outcomes should be performed in accordance with the standard operation of clinical test in each center.

(2)NEI network and specific protein test should follow the instructions by Longhua hospital.

## **6.4 Muti-center coordinating committee**

The sponsor should be in charge of building clinical coordinating committee in every center. The committees function in coordinating the trial and solve problems.

## **6.5 Monitors**

The sponsors can send monitors to ensure the trial.

# 7 Data management

## **7.1 Case report form (CRF)**

The investigators should copy and fax pp. 7-8 of the CRF (the general data, history, etc) to the Second Affiliated Hospital of Tianjin University of Traditional Chinese Medicine three days after the enrichment, in order to guarantee timely information summary analysis. Fax number: 022-60335229.

According to the original observation records of the patients, the investigators should record the date timely, correctly, completely, and clearly. CRF should be completed in three days after the patients leave. The completed CRF should also be transferred to the data administrator after checked by the monitors.

## **7.2 Data entry and modification**

Data manager is responsible for data entry and management. The dada manager defines the data range checking and logic checking content with the main researchers, according to the range of values of each index of CRF, and write program. Before inputting, control error data, and record all error content and modified results. In order to ensure th integrity of data, the data should be input by two independent data administrator.

## **7.3 Data processing**

After confirming the corrective of the database established, research administrators and statistical analysis should lock the data. The errors found after locking should be modified by the statistical analysis program after confirming the error. The database should be analyzed by the statistician, and the statistician report he data analysis to the sponsors to finish a study report.

# 8 Confidentiality

The clinical researcher should pay attention to the secrecy of the trial, and should not leak the data to others.
